# Altered gut microbial networks and metabolic pathways in multiple system atrophy: a comparative 16S rRNA study

**DOI:** 10.3389/fnins.2025.1623165

**Published:** 2025-08-13

**Authors:** Po-Chun Liu, Shao-Ying Cheng, Chih-Chi Li, Yu-Ke Wang, Yufeng Jane Tseng, Ming-Che Kuo

**Affiliations:** ^1^Cathay General Hospital, Taipei, Taiwan; ^2^Department of Neurology, National Taiwan University Hospital Bei-Hu Branch, Taipei, Taiwan; ^3^Graduate Institute of Biomedical Electronics and Bioinformatics, College of Electrical Engineering and Computer Science, National Taiwan University, Taipei, Taiwan; ^4^Department of Computer Science and Information Engineering, College of Electrical Engineering and Computer Science, National Taiwan University, Taipei, Taiwan; ^5^Department of Medicine, National Taiwan University Cancer Center, Taipei, Taiwan; ^6^College of Medicine, National Taiwan University, Taipei, Taiwan; ^7^Neurobiology and Cognitive Science Center, National Taiwan University, Taipei, Taiwan

**Keywords:** multiple system atrophy, Parkinson’s disease, gut microbiome, 16S rRNA, differential abundance analyses, correlation and network analyses

## Abstract

**Introduction:**

The alterations in the gut microbial network in multiple system atrophy (MSA) remain poorly understood. This study aimed to identify key gut microbial interaction networks in MSA through comprehensive multimodal analyses.

**Methods:**

Demographic information and frozen fecal specimens were collected from 119 participants [MSA, *n* = 26; Parkinson’s disease (PD), *n* = 66; healthy control (HC), *n* = 27]. Raw amplicons of the bacterial 16S rRNA V3–V4 gene region were processed using two methods: DADA2-denoising and clustering into operational taxonomic units. We conducted univariate and multivariable analyses to assess the differential abundance of bacterial genera and predicted metabolic pathways using four statistical methods: ANCOM, ANCOM-BC, ALDEx2, and MaAsLin 2. Interbacterial interactions were assessed using four correlation and two network analyses.

**Results:**

We consistently observed lower levels of *Fusicatenibacter* in MSA patients and lower levels of *Butyricicoccus* in PD patients compared with HCs (q < 0.05), both before and after adjusting for comorbidities, diet, and constipation status. The random forest classifiers effectively differentiated between MSA and PD, achieving high AUCs (0.75–0.78) in 5-fold cross-validation. A significant positive interbacterial interaction between *Ruminococcus gnavus* group and *Erysipelatoclostridium* was uniquely observed in MSA patients. Additionally, we identified an increase in the ARGORNPROST-PWY pathway (L-arginine degradation, q = 0.003) and a decrease in the PWY-6478 pathway (GDP-D-glycero-α-D-manno-heptose biosynthesis, q = 0.015) in MSA patients compared with HCs.

**Conclusion:**

Future studies are warranted to determine whether fecal microbiome-derived signatures can serve as reliable biomarkers for MSA.

## 1 Introduction

Multiple system atrophy (MSA) is a rare adult-onset neurodegenerative disorder characterized by glial cytoplasmic inclusions made of misfolded α-synuclein (Schrag et al., 1999; Spillantini et al., 1998). Although both MSA and Parkinson’s disease (PD) are classified as α-synucleinopathies, MSA is characterized by levodopa-unresponsive parkinsonism, autonomic dysfunction, and cerebellar ataxia (Krismer and Wenning, 2017). In contrast to PD, MSA progresses more rapidly, leading to severe disability within approximately 5 years and mortality within a decade of symptom onset (Watanabe et al., 2002; Wenning et al., 2013). While current epidemiological studies have failed to establish consistent associations between external environmental risk factors and MSA, internal environmental factors, particularly the gut microbiome, may contribute to the pathogenesis of MSA (Vanacore, 2005).

The gut microbiome plays a significant role in human health and disease through the gut-brain axis, including stimulation of immune responses and production of short-chain fatty acids (SCFAs) (Wang and Kasper, 2014). Numerous studies, including cross-sectional, longitudinal, and meta-analyses, have identified consistent alterations in the gut microbiome associated with PD, such as the enrichment of *Bifidobacterium* and the depletion of SCFA-producing bacteria like *Lachnospiraceae* and *Faecalibacterium* (Romano et al., 2021). Similar to PD, gut microbial alterations may contribute to the pathogenesis of MSA, as patients with MSA exhibit a proinflammatory colonic microbiome, impaired intestinal barrier integrity, and elevated levels of endotoxin-related intestinal inflammatory biomarkers (Engen et al., 2017). Additionally, both MSA and PD exhibit α-synuclein over-deposition (Brück et al., 2016). Dysbiosis of the gut microbiome, together with gut inflammation and hyperpermeability, can lead to α-synuclein seeding and propagation from the enteric nervous system to the central nervous system (CNS) via the vagus nerve (Klann et al., 2021). It is plausible that the gut microbiome contributes to MSA pathogenesis through the gut-brain axis.

In contrast to the growing body of evidence supporting the gut microbiome’s role in the pathogenesis and progression of PD (Menozzi et al., 2025; Tan et al., 2022), investigations in MSA have proven to be challenging because of its rare incidence and prevalence. Only few studies with limited sample sizes have examined the gut microbial composition in MSA. Furthermore, recent advancements in computational tools and bioinformatic methodologies, coupled with the lack of standardized protocols, can lead to inconsistent results in microbiome research (Bharti and Grimm, 2021). This study aimed to identify distinctive gut microbial signatures in MSA through comprehensive multimodal analyses. We employed two amplicon-preprocessing approaches and four statistical methods to enhance the robustness and reproducibility of our findings. Two statistical models were utilized to account for potential confounders, including comorbidities, diet, and constipation status. Through the identification of MSA-specific microbial fingerprints, interaction networks, and relevant metabolic pathways, we sought to advance the capabilities of differential diagnosis and enhance our understanding of the pathogenesis of MSA.

## 2 Materials and methods

### 2.1 Study subjects and collection of fecal specimens

This hospital-based case-control study was carried out at the National Taiwan University Cancer Center (NTUCC), Taipei, Taiwan, from August 2020 to December 2022. Patients meeting the criteria for clinically probable MSA according to the 2022 Movement Disorder Society criteria, as well as patients diagnosed with PD based on the United Kingdom Parkinson’s Disease Society Brain Bank clinical diagnostic criteria, were enrolled by two specialists in movement disorders (S.Y. Cheng, M.C. Kuo) (Hughes et al., 1992; Wenning et al., 2022). Each patient underwent a minimum follow-up period of 1 year. Healthy controls (HCs) were recruited from healthy spouses or caregivers of patients, or asymptomatic volunteers at the hospital. Individuals under 40 years old, with concurrent malignancies, cerebrovascular diseases, or known neurodegenerative diseases, were excluded from the study. All subjects completed a face-to-face interview with a structured, semi-quantitative questionnaire adapted from the previous study (Kuo et al., 2022). The Unified Parkinson’s Disease Rating Scale Part III (UPDRS III) score was assessed in PD patients. (Martínez-Martín et al., 2015).

Fecal specimens were collected with informed consent and with the approval of the National Taiwan University Hospital (NTUH) Research Ethics Committee (REC) (No. 202006060RINB). The specimens were stored in a sterile tube at −80°C until processing. All procedures involving human participants in this study were conducted in accordance with the ethical standards of the NTUH REC and the Declaration of Helsinki. The study was approved by the NTUCC REC.

### 2.2 DNA extraction, PCR amplification, and sequencing

Fecal DNA was extracted from 200 mg frozen samples using the QIAamp Fast DNA Stool Mini Kit (Qiagen, Hilden, Germany). The DNA samples were then stored at −20°C. The 341f forward primer and 805r reverse primer were designed to include sequences complementary to the upstream and downstream regions of the 16S rRNA V3–V4 gene segment, along with the Illumina overhang adapter sequences. The dual-index barcodes and the Illumina sequencing adapters were attached to the PCR products using the Nextera XT Index Kit (Illumina, CA, United States). The libraries were cleaned, normalized, pooled, and sequenced using the MiSeq System (Illumina, CA, United States) with V3 reagent for paired-end sequencing (2 × 300 bps).

### 2.3 Preprocessing of amplicons and taxonomic classification

The analyses of microbial composition were primarily conducted utilizing QIIME 2 2022.11 unless otherwise specified (Bolyen et al., 2019). Two distinct approaches were employed to preprocess the amplicons of 16S rRNA V3-V4 gene segment: denoising using DADA2 and *de novo* clustering into operational taxonomic units (OTUs) at a 99% identity threshold (Callahan et al., 2016; Rognes et al., 2016). The reference taxonomy was SILVA release SSU Ref NR 138.1^1^ (Quast et al., 2013). Because SILVA does not curate sequences to the species level, species-level information was excluded, and genus-level assignments may mask biologically divergent species. We assembled ecologically informed prior class weights and built a bespoke taxonomy classifier (Bokulich et al., 2018; Kaehler et al., 2019).

### 2.4 Differential abundance analyses

Multiple algorithms designed for compositional data were used to conduct differential abundance analyses across the study groups and between subjects with and without constipation. These algorithms included: (1) ANalysis of COmposition of Microbiomes (ANCOM) (Mandal et al., 2015), (2) ANalysis of COmposition of Microbiomes with Bias Correction (ANCOM-BC) (Lin and Peddada, 2020), (3) ANOVA-Like Differential Expression 2 (ALDEx2) (Fernandes et al., 2013), and (4) Microbiome Multivariable Associations with Linear Models 2 (MaAsLin 2) (Mallick et al., 2021).

ANCOM-BC and MaAsLin 2 support multivariable differential abundance analyses. We estimated the differential abundances of genera using two statistical models. Model 1 incorporated constipation status, since it is associated with gut microbial dysbiosis and can potentially lead to spurious associations in microbiome studies of human diseases, particularly in Parkinsonism (Aho et al., 2019; Vujkovic-Cvijin et al., 2020). Model 2 incorporated potential confounders that differed significantly across the study groups, including age, sex, hypertension, constipation status, medications for constipation, and usage of probiotics.

### 2.5 Correlation between MSA-associated and PD-associated genera

The ranked relative differentials estimated by Songbird were visualized using Qurro (Fedarko et al., 2020; Morton et al., 2019). Log ratios were computed between the top and bottom 25% of differentially abundant genera in comparisons of MSA vs. HC and PD vs. HC. The Student’s *t*-test was used to determine if there was a significant difference between the disease groups and HC. To investigate whether the genera that were more abundant in MSA were also more abundant in PD, and vice versa, log ratios of a genus’s mean relative abundance in MSA vs. HC were plotted against PD vs. HC.

### 2.6 Using random forest classifiers to predict disease and constipation status

To assess the potential of microbial composition as a predictor of disease status, random forest classifiers were trained. Each analysis focused on either MSA or PD, along with HC, utilizing nested 5-fold cross-validation. Each genus was assigned an importance score by the scikit-learn learning estimator (Pedregosa et al., 2011). The area under the curve (AUC) and confusion matrix were used to evaluate the predictive performance of the classifiers.

### 2.7 Microbial correlations and networks

Four statistical methods were used to calculate the microbial correlation matrices within each study group: (1) the Spearman’s correlation on centered log-ratio (CLR) transformed data, (2) SparCC, (Friedman and Alm, 2012) and Sparse Estimation of Correlations among Microbiomes (SECOM) using (3) linear correlation and (4) distance correlation (Lin et al., 2022). To ensure sufficient representation for correlation analyses while limiting the number of multiple comparisons, genera with a prevalence ≥ 50% and an average abundance ≥ 20 within each study group were included in the correlation analyses.

The inference of microbial networks was conducted using Sparse Cooccurrence Network Investigation for Compositional data (SCNIC) and SParse InversE Covariance Estimation for Ecological Association Inference (SPIEC-EASI) (Kurtz et al., 2015; Shaffer et al., 2023). Genera with an average abundance < 2 in each study group were excluded.

### 2.8 Differential pathway abundance analyses

Phylogenetic Investigation of Communities by Reconstruction of Unobserved States 2 (PICRUSt2) was used to predict the functional abundances of Enzyme Commission (EC) numbers and to regroup EC numbers into MetaCyc pathways (Caspi et al., 2016; Douglas et al., 2020). The outputs from PICRUSt2 were visualized using STAMP (Parks et al., 2014). Univariate and multivariable differential abundance analyses of MetaCyc pathways were conducted using four statistical methods (ANCOM, ANCOM-BC, ALDEx2, and MaAsLin 2).

### 2.9 Statistical analyses

The categorical variables are presented as count (percentage), while the numeric variables are presented as mean ± standard deviation (SD). The Fisher’s exact test was used to compare categorical variables across the study groups. Comparisons of age across the study groups were performed using the ANOVA with the Tukey’s post-hoc test. The Student’s *t*-test was used to compare age of onset and disease duration between MSA and PD. *P*-values < 0.05 were considered statistical significance.

The Supplementary material provided details on the microbial composition analyses. The features were collapsed at the genus level before subsequent analyses. To control false discovery rates, q values derived from the Benjamini-Hochberg procedure were used in differential abundance analyses (ANCOM, ALDEx2, and MaAsLin 2), except for ANCOM-BC, which adopted the Holm-Bonferroni method as its default approach. Q values < 0.05 were considered statistical significance. The analyses were carried out using R packages^2^ and SAS version 9.4 (SAS Institute Inc., Cary, NC, United States) (R Core Team, 2023).

## 3 Results

### 3.1 Clinicodemographic characteristics

A total of 119 participants (MSA, *n* = 26; PD, *n* = 66; HC, *n* = 27) were enrolled. After quality control, 24 MSA, 52 PD, and 27 HC samples remained after DADA2-denoising, while 24 MSA, 50 PD, and 27 HC samples remained after OTU-clustering. Table 1 presents the clinicodemographic characteristics of the study subjects with qualified fecal specimens using both approaches. The PD patients were older than the MSA patients and HCs (*p* < 0.01). In addition, a greater proportion of MSA and PD patients reported experiencing constipation, taking medications for constipation, and using probiotics compared with HCs (*p* < 0.01). The PD patients displayed mild motor symptoms according to the UPDRS III score (15.2 ± 12.0). Among PD patients, those with constipation had higher UPDRS III scores (*p* = 0.011, Supplementary Table 1).

**TABLE 1:** Clinicodemographic characteristics.

Characteristics	MSA	PD	HC	*P*-value
**No.**	24	50	27	–
**Sex**				0.058
Male	13 (54.2%)	26 (52.0%)	7 (25.9%)	–
Female	11 (45.8%)	24 (48.0%)	20 (74.1%)	–
**Age (years)**	62.0 ± 8.4	73.7 ± 8.4	67.1 ± 9.2	< 0.001 (MSA vs. HC: 0.093, PD vs. HC: 0.005, MSA vs. PD: < 0.001)
**Age (years)**				< 0.001
40–49	1 (4.2%)	0 (0.0%)	0 (0.0%)	–
50–59	5 (20.8%)	4 (8.0%)	5 (18.5%)	–
60–69	15 (62.5%)	12 (24.0%)	13 (48.2%)	–
70–79	3 (12.5%)	21 (42.0%)	7 (25.9%)	–
≥ 80	0 (0.0%)	13 (26.0%)	2 (7.4%)	–
**Hypertension**				0.042
Yes	6 (25.0%)	19 (38.0%)	16 (59.3%)	–
No	18 (75.0%)	31 (62.0%)	11 (40.7%)	–
**Diabetes mellitus**				0.606
Yes	2 (8.3%)	9 (18.0%)	3 (11.1%)	–
No	22 (91.7%)	41 (82.0%)	24 (88.9%)	–
**Hyperlipidemia**				0.124
Yes	6 (25.0%)	13 (26.0%)	13 (48.2%)	–
No	18 (75.0%)	37 (74.0%)	14 (51.9%)	–
**Cigarette smoking**				0.313
Yes	0 (0.0%)	1 (2.0%)	2 (7.4%)	–
No	24 (100.0%)	49 (98.0%)	25 (92.6%)	–
**Alcohol consumption**				1.000
Yes	0 (0.0%)	1 (2.0%)	1 (3.7%)	–
No	24 (100.0%)	49 (98.0%)	26 (96.3%)	–
**Constipation**				< 0.001
Yes	19 (79.2%)	28 (56.0%)	7 (25.9%)	–
No	5 (20.8%)	22 (44.0%)	20 (74.1%)	–
**Medications for constipation**				0.008
Yes	12 (50.0%)	17 (34.0%)	3 (11.1%)	–
No	12 (50.0%)	33 (66.0%)	24 (88.9%)	–
**Usage of probiotics**				< 0.001
Yes	12 (50.0%)	25 (50.0%)	2 (7.4%)	–
No	12 (50.0%)	25 (50.0%)	25 (92.6%)	–
**Daily vegetables**				1.000
Yes	16 (66.7%)	34 (68.0%)	19 (70.4%)	–
No	8 (33.3%)	16 (32.0%)	8 (29.6%)	–
**Daily yogurt**				1.000
Yes	1 (4.2%)	4 (8.0%)	2 (7.4%)	–
No	23 (95.8%)	46 (92.0%)	25 (92.6%)	–
**Age of onset (years)**	59.5 ± 8.1	69.6 ± 8.6	–	< 0.001
**Disease duration (years)**	2.0 ± 1.3	4.1 ± 4.6	–	0.004
**UPDRS III**	–	15.2 ± 12.0	–	–

The categorical variables are presented as count (percentage). The numeric variables are presented as mean ± SD. The Fisher’s exact test was used to compare categorical variables. The ANOVA with Tukey’s post-hoc test was used to compare age. The Student’s *t*-test was used to compare age of onset and disease duration between MSA and PD. HC, healthy control; MSA, multiple system atrophy; PD, Parkinson’s disease; UPDRS III, Unified Parkinson’s Disease Rating Scale Part III.

### 3.2 Alpha diversities and beta diversities

There were no significant differences in the observed number of features, the Shannon’s diversity index, or the Faith’s phylogenetic diversity across the study groups using either DADA2-denoising or OTU-clustering. The structures of the microbial composition are depicted in Supplementary Figure 1. A notable distinction in beta diversities, as indicated by unweighted and weighted UniFrac distances, was observed between MSA and HC in PERmutational Multivariate ANalysis of Variance (PERMANOVA) (q < 0.05, Supplementary Table 2; Anderson, 2001). On the other hand, not all beta diversity measurements between PD and HC, or between MSA and PD, were significantly different.

### 3.3 Univariate differential abundance analyses

As shown in Table 2, *Fusicatenibacter* consistently exhibited a significantly lower abundance in MSA than in HC across all four algorithms using both DADA2-denoising and OTU-clustering. Furthermore, *Butyricicoccus* was significantly less abundant in MSA than in HC when ANCOM-BC was used. On the other hand, a significant decrease in *Butyricicoccus* abundance was observed in PD compared with HC across all algorithms, except ALDEx2. When comparing MSA and PD, only ANCOM-BC identified *Limosilactobacillus* as exhibiting a significantly greater abundance in PD using DADA2-denoising (q = 0.016).

**TABLE 2:** Significant genera identified in differential abundance analyses.

	MSA vs. HC			PD vs. HC			MSA vs. PD		
**ANCOM**	**Genus**	**CLR**	**W value**	**Genus**	**CLR**	**W value**	**Genus**	**CLR**	**W value**
DADA2-denoising	***Fusicatenibacter***	−2.08	120	***Butyricicoccus***	−1.38	99	–	–	–
OTU-clustering	***Fusicatenibacter***	−1.95	209	***Butyricicoccus***	−1.38	190	–	–	–
**ANCOM-BC**	**Genus**	**LFC**	**q value**	**Genus**	**LFC**	**q value**	**Genus**	**LFC**	**q value**
DADA2-denoising	***Fusicatenibacter***	−2.08	< 0.001	***Butyricicoccus***	−1.29	0.019	*Limosilactobacillus*	−0.91	0.016
	*Faecalibacterium*	−1.85	0.024	–	–	–	–	–	–
	***Butyricicoccus***	−1.50	0.015	–	–	–	–	–	–
OTU-clustering	***Fusicatenibacter***	−1.93	< 0.001	***Butyricicoccus***	−1.43	< 0.001	–	–	–
	***Butyricicoccus***	−1.37	0.010	–	–	–	–	–	–
**ALDEx2**	**Genus**	**Effect**	**q value ^Wi^**	**Genus**	**Effect**	**q value ^Wi^**	**Genus**	**Effect**	**q value ^Wi^**
DADA2-denoising	***Fusicatenibacter***	−0.69	0.024	–	–	–	–	–	–
OTU-clustering	***Fusicatenibacter***	−0.76	0.020	–	–	–	–	–	–
**MaAsLin 2**	**Genus**	**Coefficient**	**q value**	**Genus**	**Coefficient**	**q value**	**Genus**	**Coefficient**	**q value**
DADA2-denoising	***Fusicatenibacter***	−2.26	0.013	***Butyricicoccus***	−1.76	0.008	–	–	–
OTU-clustering	***Fusicatenibacter***	−2.72	0.013	***Butyricicoccus***	−2.01	0.008	–	–	–

In ANCOM, the W value represents the number of tests showing significant differences in the ratios of a particular genus and the other genera between the two study groups. The CLR represents the difference in the means of centered log-ratio-transformed relative abundance of a genus between the two study groups. In ANCOM-BC, the LFC quantifies the effect of study group on the bias-corrected absolute abundance of a particular genus. The q values were determined using the Holm-Bonferroni method. In ALDEx2, the “effect” is determined by calculating the median difference in the CLR-transformed probabilities between the study groups and dividing it by the maximum difference in the CLR-transformed probabilities within the study groups through 128 Monte Carlo samplings. The q value for the Wilcoxon rank test was determined using the Benjamini-Hochberg procedure and is represented as q value ^Wi^. In MaAsLin 2, the “coefficient” refers to the effect size in the linear model, representing the difference between categorical variables. The q values were determined using the Benjamini-Hochberg procedure. A positive CLR/LFC/“effect”/“coefficient” suggests that the genus is more abundant in the first study group. The genera highlighted in bold were identified using both DADA2-denoising and OTU-clustering. ALDEx2, ANOVA-Like Differential Expression 2; ANCOM, ANalysis of COmposition of Microbiomes; ANCOM-BC, ANalysis of COmposition of Microbiomes with Bias Correction; CLR, centered log-ratio; HC, healthy control; LFC, log fold change; MaAsLin 2, Microbiome Multivariable Associations with Linear Models 2; MSA, multiple system atrophy; OTU, operational taxonomic unit; PD, Parkinson’s disease.

### 3.4 Multivariable differential abundance analyses

The differential abundances of genera were estimated after adjusting for potential confounders in two models. Model 1 included constipation status, while Model 2 included age, sex, hypertension, constipation status, medications for constipation, and usage of probiotics. *Fusicatenibacter* and *Butyricicoccus* remained significantly less abundant in MSA and PD, respectively, than in HC (*p* < 0.01, Supplementary Table 3). The effect size exhibited consistency with those observed in the univariate analyses.

In multivariable ANCOM-BC, *Phascolarctobacterium* exhibited a lower abundance in MSA than in HC in Model 1 using both DADA2-denoising and OTU-clustering (q < 0.05, Supplementary Table 4). In Model 2, both multivariable ANCOM-BC and multivariable MaAsLin 2 identified *Butyricicoccus* as being less abundant and *Intestinibacter* as being more abundant in MSA compared with HC (q < 0.05, Supplementary Tables 4, 5).

### 3.5 MSA-associated genera and PD-associated genera were correlated

**FIGURE 1:**
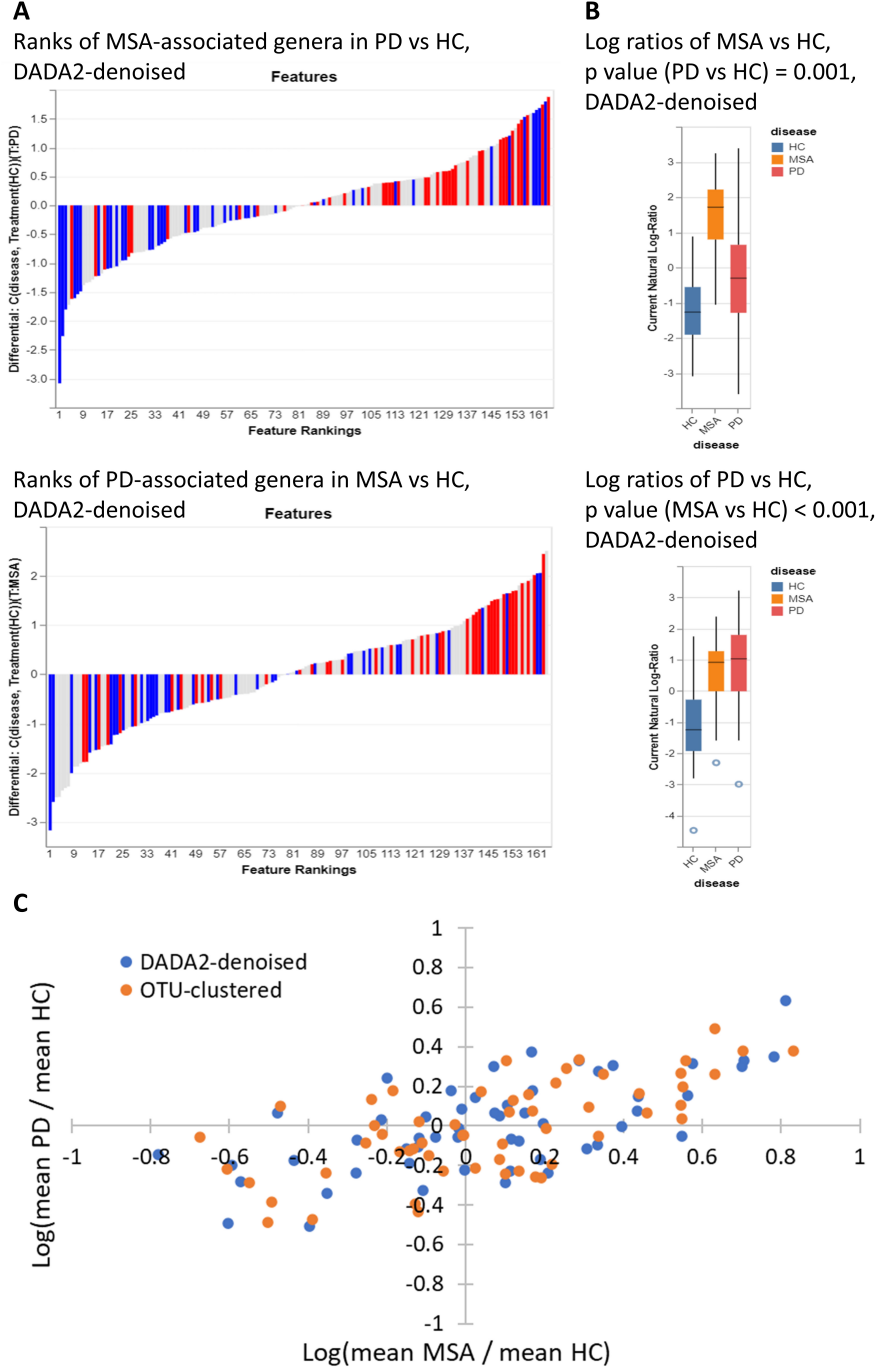
Correlation between MSA-associated genera and PD-associated genera. **(A)** Distribution of the top and bottom 25% of differentially abundant genera (MSA vs. HC) in the ranks of relative differentials between PD and HC, and vice versa. The red bars represent the top 25% most abundant genera in MSA (or PD) compared with HC, while the blue bars represent the bottom 25% least abundant genera in MSA (or PD) compared with HC. The ranking of genera was determined by their relative differentials between PD (or MSA) and HC. Songbird was employed to calculate the relative differential of each genus, and Qurro was employed to visualize the ranked relative differentials. Songbird was DADA2-denoising was used in sample preprocessing. **(B)** Log ratios between the top and bottom 25% of differentially abundant genera between MSA and HC and between PD and HC. *P*-values were determined using the Student’s *t*-test. Qurro was used to display the distributions of log ratios. DADA2-denoising was used in sample preprocessing. **(C)** Scatter plot showing the log ratios of mean relative abundances of genera (MSA vs. HC against PD vs. HC). Each dot represents a genus. The blue dots represent genera identified through DADA2-denoising, and the orange dots represent genera identified through OTU-clustering. Only genera with a mean relative abundance ≥ 0.001 across all three study groups were considered for analysis and visualization. HC, healthy control; MSA, multiple system atrophy; OTU, operational taxonomic unit; PD, Parkinson’s disease.

The ranked relative differentials are illustrated in Figure 1A. Compared with those in HC, genera exhibiting greater abundances in MSA tended to be more abundant in PD, and vice versa. At the same time, genera with a lower abundance in MSA generally displayed a reduced abundance in PD, and vice versa. Figure 1B displays the log ratios between the top and bottom 25% of differentially abundant genera between MSA and HC, as well as between PD and HC, across the three study groups. The genera that differentiated MSA from HC were capable of differentiating PD from HC, and vice versa (*p* < 0.005). Both DADA2-denoising and OTU-clustering yielded similar results (Supplementary Figure 2). Figure 1C displays the log ratios of the mean relative abundances of genera (MSA vs. HC against PD vs. HC). The Pearson correlation coefficients of the genera identified through DADA2-denoising and OTU-clustering were both 0.66 (*p* < 0.001).

### 3.6 Using random forest classifiers to predict disease status and identify genera with high importance scores

The AUCs of MSA-HC classifier and PD-HC classifier were 0.74 and 0.78 using DADA2 denoising (Supplementary Figure 3). When using OTU-clustering, the AUCs of MSA-HC classifier and PD-HC classifier were 0.67 and 0.78. The random forest classifiers built for differentiating between MSA and PD had AUCs of 0.78 and 0.75 using DADA2-denoising and OTU-clustering, respectively. Supplementary Table 6 presents the confusion matrices, which illustrate the sensitivity and specificity of the predictive performance.

Table 3 lists the genera with importance scores ≥ 0.05 in the random forest classifiers. *Fusicatenibacter* had the highest importance score in MSA-HC classifiers, while *Butyricicoccus* had the highest importance score in PD-HC classifiers. Additionally, using both DADA2-denoising and OTU-clustering, *Faecalibacterium* and *Blautia* were found to have importance scores **≥** 0.05 in MSA-HC classifiers and PD-HC classifiers, respectively. In MSA-PD classifiers, the identified genus was *Collinsella*.

**TABLE 3:** Crucial genera in random forest classifiers.

MSA-HC classifier		PD-HC classifier		MSA-PD classifier	
Genus	Importance score	Genus	Importance score	Genus	Importance score
**DADA2-denoising**					
***Fusicatenibacter***	0.066	***Butyricicoccus***	0.057	***Collinsella***	0.063
***Faecalibacterium***	0.054	***Blautia***	0.054	Uncultured genus within *Oscillospiracea*	0.055
**OTU-clustering**					
***Fusicatenibacter***	0.220	***Butyricicoccus***	0.160	***Collinsella***	0.095
***Faecalibacterium***	0.094	*Collinsella*	0.076	–	–
*Enterorhabdus*	0.053	***Blautia***	0.061	–	–
*Escherichia-Shigella*	0.052	*Eggerthella*	0.051	–	–

Genera with higher importance scores demonstrated greater efficacy in distinguishing disease status. The importance scores were determined by the scikit-learn learning estimator. Genera with importance scores ≥ 0.05 are displayed. Genera highlighted in bold were identified using both DADA2-denoising and OTU-clustering. HC, healthy control; MSA, multiple system atrophy; PD, Parkinson’s disease.

### 3.7 Microbial correlations

**FIGURE 2:**
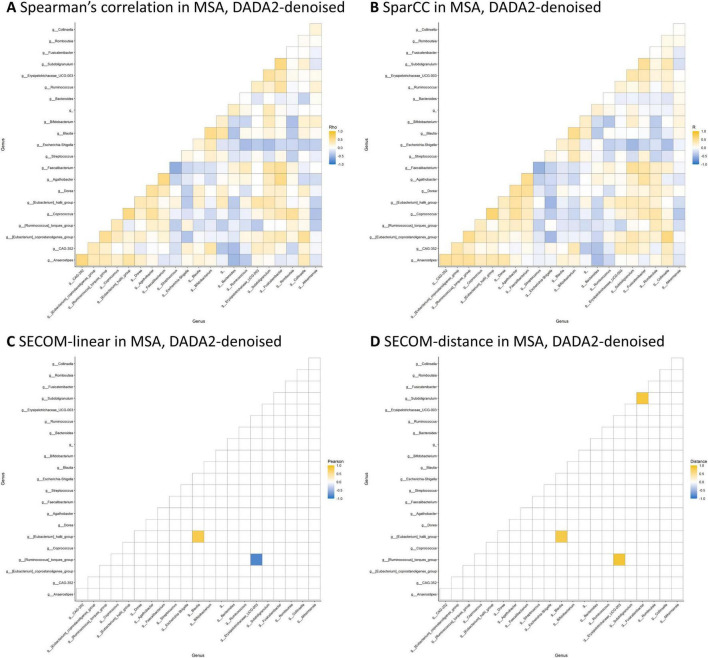
Microbial correlation matrices in MSA. Four methods were employed to calculate the microbial correlation matrices, including the Spearman’s correlation on CLR-transformed data **(A)**, SparCC **(B)**, SECOM using linear correlation **(C)**, and distance correlation **(D)**. Only genera with a prevalence ≥ 50% and an average abundance ≥ 20 in each study group were considered for analyses. The correlation coefficient, which ranges from –1 to 1, was represented using a color gradient from blue to yellow. Statistical significance is denoted by small circles following correction for multiple comparisons. The order of the genera was identical across all four matrices. DADA2-denoising was used in sample preprocessing. MSA, multiple system atrophy; SECOM, Sparse Estimation of Correlations among Microbiomes.

Table 4 presents the significant interbacterial correlations identified through the correlation analyses. Only correlations discovered using both DADA2-denoising and OTU-clustering are shown. No interbacterial correlation was found when employing the Spearman’s correlation on CLR-transformed data or SparCC after controlling for false discovery rates. On the other hand, SECOM using linear correlation and distance correlation revealed three significant correlations: (1) *Ruminococcus torques* group * *Erysipelotrichaceae UCG-003* (in MSA), (2) *Eggerthella* * *Anaerostipes* (in HC), and (3) *Butyricicoccus* * *Anaerostipes* (in HC). The microbial correlation matrices of MSA using DADA2-denoising are displayed in Figure 2. The Spearman’s correlation and SparCC exhibited similar patterns of interbacterial correlations, while SECOM produced sparse correlation matrices. SECOM revealed an additional interbacterial correlation using distance correlation compared with linear correlation in MSA. Supplementary Figures 4-1 to 4-5 present the remaining correlation matrices.

**TABLE 4:** Significant interbacterial correlations identified in correlation analyses.

MSA			HC			PD
Interbacterial correlation	Coefficient	*P*-value	Interbacterial correlation	Coefficient	*P*-value	
**Spearman’s correlation**						
–	–	–	–	–	–	–
**SparCC**						
–	–	–	–	–	–	–
**SECOM-linear**						
*Ruminococcus torques* group * *Erysipelotrichaceae UCG-003*	-0.84/-0.69	0.003/0.004	*Blautia* * *Eubacterium hallii* group	0.64/0.76	0.002/ < 0.001	–
–	–	–	*Eggerthella* * *Anaerostipes*	-0.81/-0.80	< 0.001/<0.001	–
–	–	–	Butyricicoccus * Anaerostipes	0.70/0.60	0.002/0.003	–
**SECOM-distance**						
*Ruminococcus torques* group * *Erysipelotrichaceae UCG-003*	0.90/0.77	0.005/0.004	*Eggerthella* * *Anaerostipes*	0.87/0.79	< 0.001/<0.001	–
–	–	–	*Butyricicoccus* * *Anaerostipes*	0.76/0.69	< 0.001/0.002	–

The interbacterial correlations listed above were identified using both DADA2-denoising and OTU-clustering. In SECOM-linear, the “coefficient” represents the Pearson correlation coefficient, while in SECOM-distance, the “coefficient” refers to the distance correlation coefficient. The statistics preceding and following the slashes were obtained using DADA2-denoising and OTU-clustering, respectively. HC, healthy control; MSA, multiple system atrophy; PD, Parkinson’s disease.

### 3.8 Microbial networks

**FIGURE 3:**
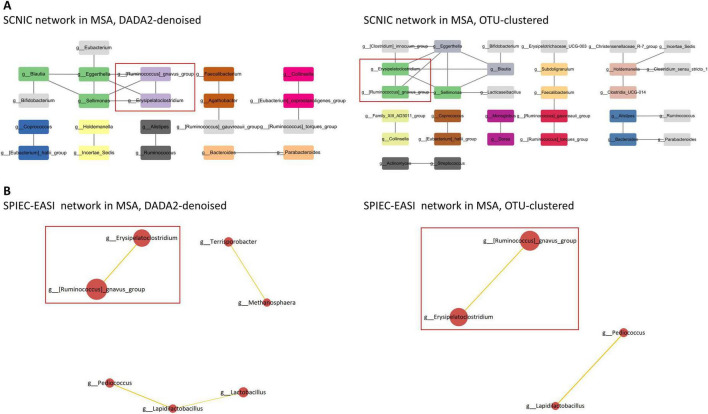
Microbial networks in MSA. **(A)** In SCNIC networks, edges are defined as correlations with an R value ≥ 0.5. The color of the nodes indicates their membership in a particular module. Visualization of the SCNIC networks was achieved using Cytoscape. The interbacterial interactions consistently identified using both SCNIC and SPIEC-EASI are highlighted. **(B)** In SPIEC-EASI networks, the node size is scaled according to the mean of CLR-transformed data, and the edge width is proportional to the absolute weight of the connection. Positive correlations are denoted by orange edges, while negative correlations are denoted by blue edges. Only edges with an absolute weight ≥ 0.1 are displayed. The interbacterial interactions consistently identified using both SCNIC and SPIEC-EASI are highlighted. MSA, multiple system atrophy; SCNIC, Sparse Cooccurrence Network Investigation for Compositional data; SPIEC-EASI, SParse InversE Covariance Estimation for Ecological Association Inference.

Significant interbacterial interactions were identified in the network analyses using both DADA2-denoising and OTU-clustering. In MSA, the positive interbacterial interaction of *Ruminococcus gnavus* group * *Erysipelatoclostridium* was consistently observed using both SCNIC and SPIEC-EASI (Figure 3 and Supplementary Table 7). This positive interaction was found to be unique to MSA, as it was not detected in the SCNIC or SPEC-EASI networks of PD and HC using either DADA2-denoising and OTU-clustering (Supplementary Figures 5–1, 5–2).

### 3.9 Differential pathway abundance analyses

**FIGURE 4:**
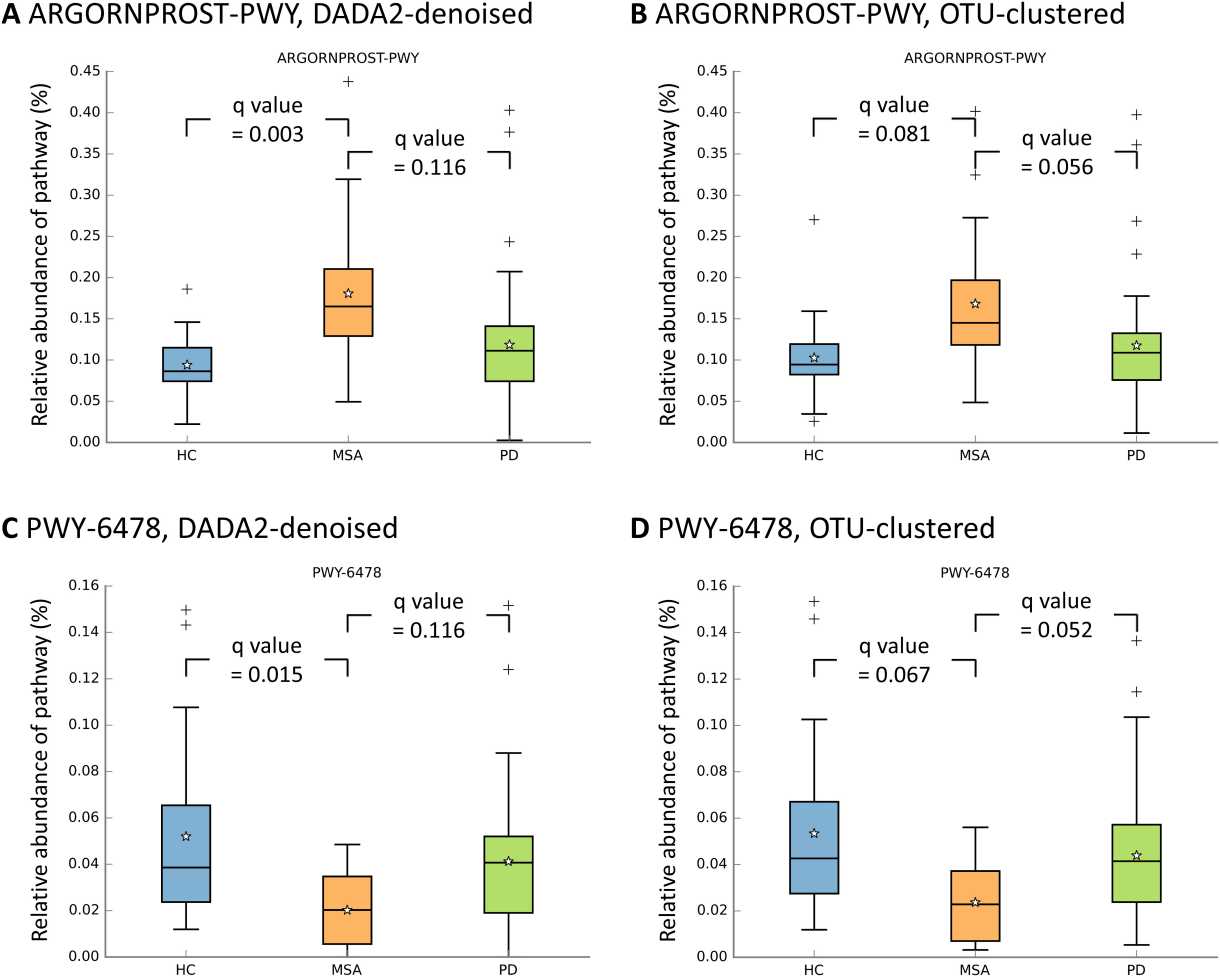
Relative abundances of metabolic pathways across study groups. The box plots illustrates the distribution of relative abundances of the ARGORNPROST-PWY pathway **(A,B)** and the PWY-6478 pathway **(C,D)**. The boxes denote the interquartile range (IQR) (25th–75th). The median value is indicated by a line inside the box, while the mean value is represented by a star. The whiskers extend to the upper and lower extreme values. The outliers are depicted as crosses. The q values obtained through the Benjamini-Hochberg procedure in univariate MaAsLin 2 are presented. HC, healthy control; MSA, multiple system atrophy; OTU, operational taxonomic unit; PD, Parkinson’s disease.

Univariate differential abundance analyses of MetaCyc pathways were conducted using four statistical methods (ANCOM, ANCOM-BC, ALDEx2, and MaAsLin 2). The ARGORNPROST-PWY pathway, a metabolic pathway leading to L-arginine degradation (Caspi et al., 2017), was significantly more abundant in MSA than in HC using DADA2-denoising across all four algorithms (Figures 4A, B and Supplementary Tables 8, 9). A similar trend was observed using OTU-clustering, although the results did not reach statistical significance. On the other hand, the PWY-6478 pathway, a metabolic pathway responsible for GDP-D-glycero-α-D-manno-heptose biosynthesis (Caspi et al., 2017), was significantly less abundant in MSA than in HC in certain algorithms (Figures 4C, D and Supplementary Tables 8, 9). When comparing MSA with PD, no pathway consistently exhibited a significantly differential abundance.

The differential abundances of MetaCyc pathways were further assessed after adjusting for potential confounders in two models. A significantly increased abundance of the ARGORNPROST-PWY pathway and a decreased abundance of the PWY-6478 pathway in MSA compared with HC were still identified using multivariable ANCOM-BC and multivariable MaAsLin 2, respectively (q < 0.05, Supplementary Tables 10, 11).

### 3.10 Constipation status was not a confounder

Because constipation may serve as a primary confounder in this study, we examined the associations between constipation status and microbial signatures. There were no significant differences in the alpha diversities between subjects with and without constipation. PERMANOVA revealed no significant differences in the beta diversities associated with constipation status (Supplementary Figures 6–1, 6–2). Furthermore, no genera exhibited a significantly differential abundance between subjects with and without constipation using either DADA2-denoising or OTU-clustering. The sensitivity analyses, including only HCs for comparison, yielded similar negative results.

Distinct genera associated with constipation status were identified in different study groups using random forest classifiers (Supplementary Table 12). The random forest classifiers for predicting constipation status in HC had AUCs of 0.77 and 0.78 using DADA2-denoising and OTU-clustering, respectively. However, when applied to MSA or PD, these classifiers exhibited limited ability to differentiate constipation status. The AUCs ranged from 0.27 to 0.57, suggesting that constipation status was associated with a distinct gut microbial shift in the disease groups compared with HC (Supplementary Table 13). In summary, the changes in gut microbiome in MSA and PD could not be attributed to constipation status.

## 4 Discussion

Previous studies have failed to consistently identify the gut microbial signatures associated with MSA and did not take potential confounders into consideration. In MSA, only higher levels of *Akkermansia* and lower levels of *Blautia* and *Faecalibacterium* were observed in more than one study (Barichella et al., 2019; Engen et al., 2017; Wan et al., 2019). Besides, only one of them directly compared the gut microbial composition and connections between MSA and PD using a simple co-abundance network analysis (Barichella et al., 2019). In this study, comprehensive multimodal analyses were conducted to reveal the altered gut microbial compositions in MSA compared with PD and HC.

To minimize the false-positive findings caused by discrepancies between the statistical methods, univariate multimodal analyses were conducted. Consistent reductions in the abundances of *Fusicatenibacter* and *Butyricicoccus* were observed in MSA and PD, respectively, in comparison with HC. Similar to other SCFA-producing bacteria, reduced levels of *Fusicatenibacter* and *Butyricicoccus* have been associated with PD (Romano et al., 2021). Studies have also shown a positive correlation between the abundance of *Butyricicoccus* and the levels of SCFAs in PD (Aho et al., 2021). Taken together, the mechanism underlying the relationship between *Fusicatenibacter* and MSA likely involves the anti-inflammatory effects of SCFAs, which is partially consistent with PD.

Only *Limosilactobacillus* exhibited a notable difference in abundance between MSA and PD in the univariate analyses. *Limosilactobacillus reuteri* is a well-studied probiotic bacterium known for its antimicrobial and immunomodulatory effects (Abuqwider et al., 2022). It can stabilize the blood-brain barrier (BBB) dysfunction associated with maternal immune activation in rodents and strengthening the gut epithelial barrier in adults (Kaur et al., 2023; Lu et al., 2023). Furthermore, a randomized controlled trial demonstrated the efficacy of a probiotic mixture containing *Limosilactobacillus reuteri* in alleviating constipation in PD (Tan et al., 2021). The lower levels of *Limosilactobacillus* in MSA compared with PD may accelerate the immune response from the gut-blood barrier to the BBB.

In multivariable analyses, *Phascolarctobacterium* exhibited lower abundances, while *Intestinibacter* exhibited higher abundances in MSA than in HC. *Phascolarctobacterium* is capable of producing propionate by fermenting succinate (Watanabe et al., 2012). Given the diverse functions of succinate, including serving as a metabolite for cross-feeding and regulating intestinal homeostasis (Fernández-Veledo and Vendrell, 2019), the associations between *Phascolarctobacterium* and neurodegenerative diseases have shown inconsistent results. On the other hand, *Intestinibacter* was found to be more abundant in children with neurodevelopmental disorders (Bojović et al., 2020). However, the relationship between *Intestinibacter* and PD has not been consistent across studies.

Although α-synuclein deposits predominate in neurons in PD, in MSA they mainly accumulate in oligodendrocytes (Spillantini et al., 1998). Distinct microbial communities in MSA versus PD may generate different signaling mediators, such as bacterial amyloidogenic proteins or metabolites like SCFAs, contributing to cell type-specific α-synuclein aggregation (Schmitt et al., 2023). SCFAs produced by the gut microbiome can modulate neuronal function and microglial maturation (Silva et al., 2020). Of note, among plasma SCFAs derived from the gut microbiome, acetic and propionic acids were decreased in MSA but increased in PD (Chen et al., 2022; He et al., 2021; Yang et al., 2022). Consequently, MSA and PD may be characterized by distinct patterns of gut microbial dysbiosis that lead to divergent plasma SCFA levels, ultimately affecting different neuronal populations and driving disease-specific α-synuclein pathology.

We further conducted an ensemble learning method using random forest classifiers to leverage the comprehensive gut microbial composition to differentiate between MSA, PD, and HC. The AUCs of the random forest classifiers ranged from 0.75 to 0.78, suggesting the potential utility of the gut microbiome in differentiating MSA from PD. *Collinsella*, which had importance scores > 0.05 in MSA-PD classifiers, is capable of producing SCFAs (Kageyama and Benno, 2015). *Collinsella* exhibits a greater abundance in numerous neurodegenerative diseases, including Alzheimer’s disease (AD) (Cammann et al., 2023), dementia with Lewy bodies (DLB) (Nishiwaki et al., 2022), and early PD (Huang et al., 2023). Notably, *Collinsella* was also found to increase with the stage and duration of PD (Zhang et al., 2020). Although the random forest classifiers based on the gut microbiome could differentiate MSA from PD, it was difficult to trace exactly how a prediction was made, limiting their direct clinical application. Furthermore, external validation in independent cohorts is needed to confirm common gut microbial alterations across populations.

It is debatable whether a single bacterial genus can significantly impact the host’s health status. Therefore, we utilized correlation analyses and network analyses to investigate the underlying structure of gut microbial compositions across the study groups. In MSA, a positive correlation between *Ruminococcus torques* group and *Erysipelotrichaceae UCG-00*3 was identified in SECOM using both linear and distance correlation. Additionally, a positive interaction between *Ruminococcus gnavus* group and *Erysipelatoclostridium* was observed in SCNIC and SPIEC-EASI networks of MSA. *Ruminococcus torques* and *Ruminococcus gnavus* are mucosa-associated bacteria that can produce SCFAs by degrading mucin (Crost et al., 2023). These bacteria may contribute to the pathogenesis of inflammatory bowel disease by either providing substrates for non-mucolytic bacteria or producing proinflammatory polysaccharides (Wiredu Ocansey et al., 2023). Furthermore, an increased abundance of *Ruminococcus torques* has been observed in DLB and multiple sclerosis (Nishiwaki et al., 2022; Thirion et al., 2023). On the other hand, *Erysipelotrichaceae UCG-003* and *Erysipelatoclostridium* have been shown to exhibit increased abundances in PD (Lubomski et al., 2022; Rosario et al., 2021). *Erysipelatoclostridium* was also positively related to the severity of PD (Papić et al., 2022). It is plausible that an interwoven metabolic interaction between these coupled bacteria is responsible for the observed positive correlations in MSA.

Notably, functional inference using PICRUSt2 predicted a significant increase in the abundance of the ARGORNPROST-PWY pathway and a significant decrease in the abundance of the PWY-6478 pathway in MSA compared with HC. The ARGORNPROST-PWY pathway leads to the degradation of L-arginine, the primary substrate for nitric oxide (NO) synthesis (Caspi et al., 2017). NO is an important regulator of the CNS, promoting optimal cerebral blood flow, regulating synaptic plasticity, and modulating neurosecretion (Virarkar et al., 2013). A previous study reported decreased nitrate levels, the degradation product of NO, in the cerebrospinal fluid of MSA patients, indicating reduced CNS production of NO (Kuiper et al., 1994). L-arginine itself can act as an immunosuppressive agent. In a rat model of cerebral ischemia-reperfusion injury, L-arginine inhibits the microglial inflammatory response, thereby exerting a neuroprotective effect (Chen et al., 2020). In PD and AD, agmatine, a metabolite of L-arginine, can reduce oxidative stress and decreases neuronal apoptosis by inhibiting excitatory amino acid-induced neurotoxicity (Xu et al., 2018). L-arginine also inhibits the aggregation of amyloidogenic β-sheet structures by interacting with the hydrophobic regions of proteins (Das et al., 2007). Taken together, the accelerated degradation of L-arginine may contribute to the susceptibility to α-synuclein aggregation in MSA.

On the other hand. the PWY-6478 pathway is responsible for the GDP-D-glycero-α-D-manno-heptose biosynthesis (Caspi et al., 2017). Heptoses are common components of proinflammatory lipopolysaccharides (LPSs) present on the surface of gram-negative bacterial cells (Holst et al., 1991). Gut microbial dysbiosis can lead to excessive leakage of LPS from the gut lining. LPSs primarily bind to toll-like receptor 4 (TLR4) on immune cells, causing excessive production of proinflammatory cytokines. Chronic neuroinflammation and neuronal cell death elicited via the gut-brain axis are key components of neurodegenerative diseases (Kalyan et al., 2022). MSA patients have been shown to exhibit increased TLR4 expression in their colonic mucosa (Engen et al., 2017). Despite TLR4’s role in the secretion of inflammatory mediators, it may also promote intestinal homeostasis and is essential for protection against epithelial injury and bacterial invasion (Craig et al., 2023). In our study, the decreased abundance of the PWY-6478 pathway in MSA may be associated with downregulation of intestinal TLR4 and a reduced reparative response to intestinal injury. More studies are needed to elucidate the roles of LPSs and TLR4 in the gut environment of MSA. Despite a moderate correlation between the genera associated with MSA and PD, investigations into the underlying metabolic mechanisms deserve more attention.

Our study has several limitations. First, the sample size was relatively small due to the rarity of MSA, which limited the statistical power to detect additional genera and metabolic pathways associated with MSA and PD. Furthermore, the limited number of subjects precluded the use of statistical matching techniques; therefore, we conducted multivariable analyses to control for potential confounders. Second, the associations between diseases and changes in the gut microbiome were established through the study’s cross-sectional design. Further longitudinal studies are needed to determine whether the identified microbial shifts are upstream, downstream, or incidental to MSA pathogenesis. Although the comprehensive multimodal analyses lend our study relatively robust internal validity, open datasets for external validation are needed to confirm these gut microbiome-derived signatures. We carefully controlled for potential confounders in the statistical analyses. Nonetheless, information on constipation status and daily intake of vegetables and yogurt was self-reported and assessed using a subjective true/false question, which may have introduced bias. Finally, while statistical tests did not show a significant influence of constipation on the observed microbial differences in MSA and PD, constipation may still exert residual confounding effects, as certain dysbiosis could be exclusively related to constipation in MSA patients.

In summary, our study provides the first evidence of pivotal alterations in gut microbial networks and metabolic pathways specific to MSA. Through comprehensive multimodal analyses, consistent findings were identified, particularly the decreased abundance of *Fusicatenibacter* in MSA. Although the genera associated with MSA and PD showed a moderate correlation, our correlation and network analyses revealed unique interbacterial interactions specific to MSA, including a novel positive correlation between *Ruminococcus gnavus* group and *Erysipelatoclostridium*. Further studies with larger cohorts are needed to establish the clinical utility of fecal microbiome-derived signatures that incorporate interbacterial interactions in MSA. Furthermore, we identified significantly altered metabolic pathways in MSA, such as an increase in the ARGORNPROST-PWY pathway and a decrease in the PWY-6478 pathway. Experimental validation, such as shotgun metagenomic sequencing, should be performed to validate these functional predictions. This provides new directions for future studies on microbial metabolic interactions. Our findings may contribute to the differential diagnosis of MSA and enhance our understanding of its underlying pathogenesis.

## Data Availability

The datasets presented in this study can be found in the Sequence Read Archive under the accession number PRJNA1289896.

## Footnotes

1
https://www.arb-silva.de/projects/ssu-ref-nr/

2
www.R-project.org/

## References

[B1] Abuqwider J. Altamimi M. Mauriello G. (2022). Limosilactobacillus reuteri in health and disease. *Microorganisms* 10:522. 10.3390/microorganisms10030522 35336098 PMC8953724

[B2] Aho V. T. E. Houser M. C. Pereira P. A. B. Chang J. Rudi K. Paulin L. (2021). Relationships of gut microbiota, short-chain fatty acids, inflammation, and the gut barrier in Parkinson’s disease. *Mol. Neurodegener.* 16:6. 10.1186/s13024-021-00427-6 33557896 PMC7869249

[B3] Aho V. T. E. Pereira P. A. B. Voutilainen S. Paulin L. Pekkonen E. Auvinen P. (2019). Gut microbiota in Parkinson’s disease: Temporal stability and relations to disease progression. *EBioMedicine* 44 691–707. 10.1016/j.ebiom.2019.05.064 31221587 PMC6606744

[B4] Anderson M. J. (2001). A new method for non-parametric multivariate analysis of variance. *Aus. Ecol.* 26 32–46. 10.1111/j.1442-9993.2001.01070.pp.x

[B5] Barichella M. Severgnini M. Cilia R. Cassani E. Bolliri C. Caronni S. (2019). Unraveling gut microbiota in Parkinson’s disease and atypical parkinsonism. *Mov. Disord.* 34 396–405. 10.1002/mds.27581 30576008

[B6] Bharti R. Grimm D. G. (2021). Current challenges and best-practice protocols for microbiome analysis. *Brief. Bioinform.* 22 178–193. 10.1093/bib/bbz155 31848574 PMC7820839

[B7] Bojović K. Ignjatović ÐI. Soković Bajić S. Vojnović Milutinović D. Tomić M. Golić N. (2020). Gut microbiota dysbiosis associated with altered production of short chain fatty acids in children with neurodevelopmental disorders. *Front. Cell Infect. Microbiol.* 10:223. 10.3389/fcimb.2020.00223 32509596 PMC7248180

[B8] Bokulich N. A. Kaehler B. D. Rideout J. R. Dillon M. Bolyen E. Knight R. (2018). Optimizing taxonomic classification of marker-gene amplicon sequences with QIIME 2’s q2-feature-classifier plugin. *Microbiome* 6:90. 10.1186/s40168-018-0470-z 29773078 PMC5956843

[B9] Bolyen E. Rideout J. R. Dillon M. R. Bokulich N. A. Abnet C. C. Al-Ghalith G. A. (2019). Reproducible, interactive, scalable and extensible microbiome data science using QIIME 2. *Nat. Biotechnol.* 37 852–857. 10.1038/s41587-019-0209-9 31341288 PMC7015180

[B10] Brück D. Wenning G. K. Stefanova N. Fellner L. (2016). Glia and alpha-synuclein in neurodegeneration: A complex interaction. *Neurobiol. Dis.* 85 262–274. 10.1016/j.nbd.2015.03.003 25766679 PMC4730552

[B11] Callahan B. J. McMurdie P. J. Rosen M. J. Han A. W. Johnson A. J. Holmes S. P. (2016). DADA2: High-resolution sample inference from Illumina amplicon data. *Nat. Methods* 13 581–583. 10.1038/nmeth.3869 27214047 PMC4927377

[B12] Cammann D. Lu Y. Cummings M. J. Zhang M. L. Cue J. M. Do J. (2023). Genetic correlations between Alzheimer’s disease and gut microbiome genera. *Sci. Rep.* 13:5258. 10.1038/s41598-023-31730-5 37002253 PMC10066300

[B13] Caspi R. Billington R. Ferrer L. Foerster H. Fulcher C. A. Keseler I. M. (2016). The MetaCyc database of metabolic pathways and enzymes and the BioCyc collection of pathway/genome databases. *Nucleic Acids Res.* 44 D471–D480. 10.1093/nar/gkv1164 26527732 PMC4702838

[B14] Caspi R. Billington R. Fulcher C. A. Keseler I. M. Kothari A. Krummenacker M. (2017). The MetaCyc database of metabolic pathways and enzymes. *Nucleic Acids Res.* 46 D633–D639. 10.1093/nar/gkx935 29059334 PMC5753197

[B15] Chen S. F. Pan M. X. Tang J. C. Cheng J. Zhao D. Zhang Y. (2020). Arginine is neuroprotective through suppressing HIF-1α/LDHA-mediated inflammatory response after cerebral ischemia/reperfusion injury. *Mol. Brain* 13:63. 10.1186/s13041-020-00601-9 32321555 PMC7175589

[B16] Chen S. J. Chen C. C. Liao H. Y. Lin Y. T. Wu Y. W. Liou J. M. (2022). Association of fecal and plasma levels of short-chain fatty acids with gut microbiota and clinical severity in patients with Parkinson disease. *Neurology* 98 e848–e858. 10.1212/WNL.0000000000013225 34996879 PMC8883514

[B17] Craig C. F. Finkelstein D. I. McQuade R. M. Diwakarla S. (2023). Understanding the potential causes of gastrointestinal dysfunctions in multiple system atrophy. *Neurobiol. Dis.* 187:106296. 10.1016/j.nbd.2023.106296 37714308

[B18] Crost E. H. Coletto E. Bell A. Juge N. (2023). Ruminococcus gnavus: Friend or foe for human health. *FEMS Microbiol. Rev.* 47:fuad014. 10.1093/femsre/fuad014 37015876 PMC10112845

[B19] Das U. Hariprasad G. Ethayathulla A. S. Manral P. Das T. K. Pasha S. (2007). Inhibition of protein aggregation: Supramolecular assemblies of arginine hold the key. *PLoS One* 2:e1176. 10.1371/journal.pone.0001176 18000547 PMC2064962

[B20] Douglas G. M. Maffei V. J. Zaneveld J. R. Yurgel S. N. Brown J. R. Taylor C. M. (2020). PICRUSt2 for prediction of metagenome functions. *Nat. Biotechnol.* 38 685–688. 10.1038/s41587-020-0548-6 32483366 PMC7365738

[B21] Engen P. A. Dodiya H. B. Naqib A. Forsyth C. B. Green S. J. Voigt R. M. (2017). The potential role of gut-derived inflammation in multiple system atrophy. *J. Parkinsons Dis.* 7 331–346. 10.3233/JPD-160991 28234259

[B22] Fedarko M. W. Martino C. Morton J. T. González A. Rahman G. Marotz C. A. (2020). Visualizing ’omic feature rankings and log-ratios using Qurro. *NAR Genom. Bioinform.* 2:lqaa023. 10.1093/nargab/lqaa023 32391521 PMC7194218

[B23] Fernandes A. D. Macklaim J. M. Linn T. G. Reid G. Gloor G. B. (2013). ANOVA-like differential expression (ALDEx) analysis for mixed population RNA-Seq. *PLoS One* 8:e67019. 10.1371/journal.pone.0067019 23843979 PMC3699591

[B24] Fernández-Veledo S. Vendrell J. (2019). Gut microbiota-derived succinate: Friend or foe in human metabolic diseases? *Rev. Endocrine Metab. Disord.* 20 439–447. 10.1007/s11154-019-09513-z 31654259 PMC6938788

[B25] Friedman J. Alm E. J. (2012). Inferring correlation networks from genomic survey data. *PLoS Comput. Biol.* 8:e1002687. 10.1371/journal.pcbi.1002687 23028285 PMC3447976

[B26] He X. Qian Y. Xu S. Zhang Y. Mo C. Guo W. (2021). Plasma short-chain fatty acids differences in multiple system atrophy from Parkinson’s disease. *J. Parkinsons Dis.* 11 1167–1176. 10.3233/JPD-212604 33935107

[B27] Holst O. Zähringer U. Brade H. Zamojski A. (1991). Structural analysis of the heptose/hexose region of the lipopolysaccharide from *Escherichia coli* K-12 strain W3100. *Carbohydr. Res.* 215 323–335. 10.1016/0008-6215(91)84031-9 1794130

[B28] Huang B. Chau S. W. H. Liu Y. Chan J. W. Y. Wang J. Ma S. L. (2023). Gut microbiome dysbiosis across early Parkinson’s disease, REM sleep behavior disorder and their first-degree relatives. *Nat. Commun.* 14:2501. 10.1038/s41467-023-38248-4 37130861 PMC10154387

[B29] Hughes A. J. Daniel S. E. Kilford L. Lees A. J. (1992). Accuracy of clinical diagnosis of idiopathic Parkinson’s disease: A clinico-pathological study of 100 cases. *J. Neurol. Neurosurg. Psychiatry* 55 181–184. 10.1136/jnnp.55.3.181 1564476 PMC1014720

[B30] Kaehler B. D. Bokulich N. A. McDonald D. Knight R. Caporaso J. G. Huttley G. A. (2019). Species abundance information improves sequence taxonomy classification accuracy. *Nat. Commun.* 10:4643. 10.1038/s41467-019-12669-6 31604942 PMC6789115

[B31] Kageyama A. Benno Y. (2015). *Collinsella. Bergey’s manual of systematics of archaea and bacteria.* Hoboken, NJ: Wiley.

[B32] Kalyan M. Tousif A. H. Sonali S. Vichitra C. Sunanda T. Praveenraj S. S. (2022). Role of endogenous lipopolysaccharides in neurological disorders. *Cells* 11:4038. 10.3390/cells11244038 36552802 PMC9777235

[B33] Kaur S. Sharma P. Mayer M. J. Neuert S. Narbad A. Kaur S. (2023). Beneficial effects of GABA-producing potential probiotic *Limosilactobacillus fermentum* L18 of human origin on intestinal permeability and human gut microbiota. *Microb. Cell Fact.* 22:256. 10.1186/s12934-023-02264-2 38087304 PMC10717626

[B34] Klann E. M. Dissanayake U. Gurrala A. Farrer M. Shukla A. W. Ramirez-Zamora A. (2021). The gut-brain axis and its relation to Parkinson’s disease: A review. *Front. Aging Neurosci.* 13:782082. 10.3389/fnagi.2021.782082 35069178 PMC8776990

[B35] Krismer F. Wenning G. K. (2017). Multiple system atrophy: Insights into a rare and debilitating movement disorder. *Nat. Rev. Neurol.* 13 232–243. 10.1038/nrneurol.2017.26 28303913

[B36] Kuiper M. A. Visser J. J. Bergmans P. L. Scheltens P. Wolters E. C. (1994). Decreased cerebrospinal fluid nitrate levels in Parkinson’s disease, Alzheimer’s disease and multiple system atrophy patients. *J. Neurol. Sci.* 121 46–49. 10.1016/0022-510x(94)90155-4 8133311

[B37] Kuo M. C. Lu Y. C. Tai C. H. Soong B. W. Hu F. C. Chen M. L. (2022). COQ2 and SNCA polymorphisms interact with environmental factors to modulate the risk of multiple system atrophy and subtype disposition. *Eur. J. Neurol.* 29 2956–2966. 10.1111/ene.15475 35748722

[B38] Kurtz Z. D. Müller C. L. Miraldi E. R. Littman D. R. Blaser M. J. Bonneau R. A. (2015). Sparse and compositionally robust inference of microbial ecological networks. *PLoS Comput. Biol.* 11:e1004226. 10.1371/journal.pcbi.1004226 25950956 PMC4423992

[B39] Lin H. Peddada S. D. (2020). Analysis of compositions of microbiomes with bias correction. *Nat. Commun.* 11:3514. 10.1038/s41467-020-17041-7 32665548 PMC7360769

[B40] Lin H. Eggesbø M. Peddada S. D. (2022). Linear and nonlinear correlation estimators unveil undescribed taxa interactions in microbiome data. *Nat. Commun.* 13:4946. 10.1038/s41467-022-32243-x 35999204 PMC9399263

[B41] Lu J. Fan X. Lu L. Yu Y. Markiewicz E. Little J. C. (2023). *Limosilactobacillus reuteri* normalizes blood-brain barrier dysfunction and neurodevelopment deficits associated with prenatal exposure to lipopolysaccharide. *Gut Microbes* 15:2178800. 10.1080/19490976.2023.2178800 36799469 PMC9980478

[B42] Lubomski M. Xu X. Holmes A. J. Muller S. Yang J. Y. H. Davis R. L. (2022). The gut microbiome in Parkinson’s disease: A longitudinal study of the impacts on disease progression and the use of device-assisted therapies. *Front. Aging Neurosci.* 14:875261. 10.3389/fnagi.2022.875261 35656540 PMC9152137

[B43] Mallick H. Rahnavard A. McIver L. J. Ma S. Zhang Y. Nguyen L. H. (2021). Multivariable association discovery in population-scale meta-omics studies. *PLoS Comput. Biol.* 17:e1009442. 10.1371/journal.pcbi.1009442 34784344 PMC8714082

[B44] Mandal S. Van Treuren W. White R. A. Eggesbø M. Knight R. Peddada S. D. (2015). Analysis of composition of microbiomes: A novel method for studying microbial composition. *Microb. Ecol. Health Dis.* 26:27663. 10.3402/mehd.v26.27663 26028277 PMC4450248

[B45] Martínez-Martín P. Rodríguez-Blázquez C. Mario Alvarez, Arakaki T. Arillo V. C. Chaná P. (2015). Parkinson’s disease severity levels and MDS-Unified Parkinson’s disease rating scale. *Parkinson. Relat. Disord.* 21 50–54. 10.1016/j.parkreldis.2014.10.026 25466406

[B46] Menozzi E. Schapira A. H. V. Borghammer P. (2025). The gut-brain axis in Parkinson disease: Emerging concepts and therapeutic implications. *Mov. Disord. Clin. Pract.* 10.1002/mdc3.70029 [Epub ahead of print]. 40079755 PMC12275011

[B47] Morton J. T. Marotz C. Washburne A. Silverman J. Zaramela L. S. Edlund A. (2019). Establishing microbial composition measurement standards with reference frames. *Nat. Commun.* 10:2719. 10.1038/s41467-019-10656-5 31222023 PMC6586903

[B48] Nishiwaki H. Ueyama J. Kashihara K. Ito M. Hamaguchi T. Maeda T. (2022). Gut microbiota in dementia with Lewy bodies. *NPJ Parkinsons. Dis.* 8:169. 10.1038/s41531-022-00428-2 36494405 PMC9734655

[B49] Papić E. Rački V. Hero M. Tomić Z. Starčević-Čižmarević N. Kovanda A. (2022). The effects of microbiota abundance on symptom severity in Parkinson’s disease: A systematic review. *Front. Aging Neurosci.* 14:1020172. 10.3389/fnagi.2022.1020172 36570528 PMC9772822

[B50] Parks D. H. Tyson G. W. Hugenholtz P. Beiko R. G. (2014). STAMP: Statistical analysis of taxonomic and functional profiles. *Bioinformatics* 30 3123–3124. 10.1093/bioinformatics/btu494 25061070 PMC4609014

[B51] Pedregosa F. Varoquaux G. Gramfort A. Michel V. Thirion B. Grisel O. (2011). Scikit-learn: Machine learning in Python. *J. Mach. Learn. Res.* 12 2825–2830.

[B52] Quast C. Pruesse E. Yilmaz P. Gerken J. Schweer T. Yarza P. (2013). The SILVA ribosomal RNA gene database project: Improved data processing and web-based tools. *Nucleic Acids Res.* 41 D590–D596. 10.1093/nar/gks1219 23193283 PMC3531112

[B53] R Core Team (2023). *R: A language and environment for statistical computing. R foundation for statistical computing.* Vienna: R Core Team.

[B54] Rognes T. Flouri T. Nichols B. Quince C. Mahé F. (2016). VSEARCH: A versatile open source tool for metagenomics. *PeerJ* 4:e2584. 10.7717/peerj.2584 27781170 PMC5075697

[B55] Romano S. Savva G. M. Bedarf J. R. Charles I. G. Hildebrand F. Narbad A. (2021). Meta-analysis of the Parkinson’s disease gut microbiome suggests alterations linked to intestinal inflammation. *NPJ Parkinsons Dis.* 7:27. 10.1038/s41531-021-00156-z 33692356 PMC7946946

[B56] Rosario D. Bidkhori G. Lee S. Bedarf J. Hildebrand F. Le Chatelier E. (2021). Systematic analysis of gut microbiome reveals the role of bacterial folate and homocysteine metabolism in Parkinson’s disease. *Cell Rep.* 34:108807. 10.1016/j.celrep.2021.108807 33657381

[B57] Schmitt V. Masanetz R. K. Weidenfeller M. Ebbinghaus L. S. Süß P. Rosshart S. P. (2023). Gut-to-brain spreading of pathology in synucleinopathies: A focus on molecular signalling mediators. *Behav. Brain Res.* 452:114574. 10.1016/j.bbr.2023.114574 37423320

[B58] Schrag A. Ben-Shlomo Y. Quinn N. P. (1999). Prevalence of progressive supranuclear palsy and multiple system atrophy: A cross-sectional study. *Lancet* 354 1771–1775. 10.1016/s0140-6736(99)04137-9 10577638

[B59] Shaffer M. Thurimella K. Sterrett J. D. Lozupone C. A. (2023). SCNIC: Sparse correlation network investigation for compositional data. *Mol. Ecol. Resour.* 23 312–325. 10.1111/1755-0998.13704 36001047 PMC9744196

[B60] Silva Y. P. Bernardi A. Frozza R. L. (2020). The role of short-chain fatty acids from gut microbiota in gut-brain communication. *Front. Endocrinol.* 11:25. 10.3389/fendo.2020.00025 32082260 PMC7005631

[B61] Spillantini M. G. Crowther R. A. Jakes R. Cairns N. J. Lantos P. L. Goedert M. (1998). Filamentous alpha-synuclein inclusions link multiple system atrophy with Parkinson’s disease and dementia with Lewy bodies. *Neurosci. Lett.* 251 205–208. 10.1016/s0304-3940(98)00504-7 9726379

[B62] Tan A. H. Lim S. Y. Lang A. E. (2022). The microbiome-gut-brain axis in Parkinson disease - from basic research to the clinic. *Nat. Rev. Neurol.* 18 476–495. 10.1038/s41582-022-00681-2 35750883

[B63] Tan A. H. Lim S. Y. Chong K. K. A Manap M. A. A. Hor J. W. Lim J. L. (2021). Probiotics for constipation in parkinson disease: A randomized placebo-controlled study. *Neurology* 96 e772–e782. 10.1212/WNL.0000000000010998 33046607

[B64] Thirion F. Sellebjerg F. Fan Y. Lyu L. Hansen T. H. Pons N. (2023). The gut microbiota in multiple sclerosis varies with disease activity. *Genome Med.* 15:1. 10.1186/s13073-022-01148-1 36604748 PMC9814178

[B65] Vanacore N. (2005). Epidemiological evidence on multiple system atrophy. *J. Neural Transm.* 112 1605–1612. 10.1007/s00702-005-0380-7 16284906

[B66] Virarkar M. Alappat L. Bradford P. G. Awad A. B. (2013). L-arginine and nitric oxide in CNS function and neurodegenerative diseases. *Crit. Rev. Food Sci. Nutr.* 53 1157–1167. 10.1080/10408398.2011.573885 24007420

[B67] Vujkovic-Cvijin I. Sklar J. Jiang L. Natarajan L. Knight R. Belkaid Y. (2020). Host variables confound gut microbiota studies of human disease. *Nature* 587 448–454. 10.1038/s41586-020-2881-9 33149306 PMC7677204

[B68] Wan L. Zhou X. Wang C. Chen Z. Peng H. Hou X. (2019). Alterations of the gut microbiota in multiple system atrophy patients. *Front. Neurosci.* 13:1102. 10.3389/fnins.2019.01102 31680836 PMC6813281

[B69] Wang Y. Kasper L. H. (2014). The role of microbiome in central nervous system disorders. *Brain Behav. Immun.* 38 1–12. 10.1016/j.bbi.2013.12.015 24370461 PMC4062078

[B70] Watanabe H. Saito Y. Terao S. Ando T. Kachi T. Mukai E. (2002). Progression and prognosis in multiple system atrophy: An analysis of 230 Japanese patients. *Brain* 125(Pt 5), 1070–1083. 10.1093/brain/awf117 11960896

[B71] Watanabe Y. Nagai F. Morotomi M. (2012). Characterization of *Phascolarctobacterium succinatutens* sp. nov., an asaccharolytic, succinate-utilizing bacterium isolated from human feces. *Appl. Environ. Microbiol.* 78 511–518. 10.1128/AEM.06035-11 22081579 PMC3255759

[B72] Wenning G. K. Geser F. Krismer F. Seppi K. Duerr S. Boesch S. (2013). The natural history of multiple system atrophy: A prospective European cohort study. *Lancet Neurol.* 12 264–274. 10.1016/S1474-4422(12)70327-7 23391524 PMC3581815

[B73] Wenning G. K. Stankovic I. Vignatelli L. Fanciulli A. Calandra-Buonaura G. Seppi K. (2022). The movement disorder society criteria for the diagnosis of multiple system atrophy. *Mov. Disord.* 37 1131–1148. 10.1002/mds.29005 35445419 PMC9321158

[B74] Wiredu Ocansey D. K. Hang S. Yuan X. Qian H. Zhou M. Valerie Olovo C. (2023). The diagnostic and prognostic potential of gut bacteria in inflammatory bowel disease. *Gut Microbes* 15:2176118. 10.1080/19490976.2023.2176118 36794838 PMC9980661

[B75] Xu W. Gao L. Li T. Shao A. Zhang J. (2018). Neuroprotective role of agmatine in neurological diseases. *Curr. Neuropharmacol.* 16 1296–1305. 10.2174/1570159X15666170808120633 28786346 PMC6251039

[B76] Yang X. Ai P. He X. Mo C. Zhang Y. Xu S. (2022). Parkinson’s disease is associated with impaired gut-blood barrier for short-chain fatty acids. *Mov. Disord.* 37 1634–1643. 10.1002/mds.29063 35607987

[B77] Zhang F. Yue L. Fang X. Wang G. Li C. Sun X. (2020). Altered gut microbiota in Parkinson’s disease patients/healthy spouses and its association with clinical features. *Parkinson. Relat. Disord.* 81 84–88. 10.1016/j.parkreldis.2020.10.034 33099131

