# Supplementary material for: Altered gut microbial networks and metabolic pathways in multiple system atrophy: a comparative 16S rRNA study
Source: Front Neurosci. 2025 Aug 13;19:1623165. doi: 10.3389/fnins.2025.1623165 (PMC12380817; doi:10.3389/fnins.2025.1623165)
Supplement: Supplementary file 1 [file Data_Sheet_1.pdf]

Supplementary Material

1 Supplementary Data

1.1 PCR amplification

The DNA samples were standardized to a concentration of 10 ng/µL before PCR amplification. The sequences of the PCR primers were as follows:

5'TCGTCGGCAGCGTCAGATGTGTATAAGAGACAGCCTACGGGNGGCWGCAG

(341f forward primer) and

5' GTCTCGTGGGCTCGGAGATGTGTATAAGAGACAGGACTACHVGGGTATCTAATCC

(805r reverse primer). The regions underlined in the sequences represent the complementary regions.

The thermal cycling parameters for the PCR process involved an initial denaturation step at 95 °C for 3 minutes, followed by 28 cycles of denaturation at 95 °C for 30 seconds, annealing at 55 °C for 30 seconds, and extension at 72 °C for 30 seconds, and a final extension step at 72 °C for 5 minutes. Subsequently, the PCR products were purified using AMPure XP Beads (Beckman Coulter, CA, USA).

1.2 Importing raw data

The analyses of microbial composition were primarily conducted utilizing QIIME 2 2022.11 within the conda environment unless otherwise specified.(1) The raw data consisted of FASTQ files containing processed amplicons from which sequencing adapters, dual-index barcodes, and overhang adapters were removed. The forward and reverse complementary sequences of the amplicons were trimmed, and reads lacking the complementary sequences were discarded. One sample, PD15, was excluded from the analyses because it had read counts < 100. Following demultiplexing,(2) the median number of reads was 3,260 (ranging from 764 to 5,515). The sequencing quality is depicted in Figure 1.

1.3 Denoising reads using DADA2

Ideally, the PCR primers were designed to generate amplicons of 465 nucleotides (nt) (805 - 341

+ 1). Removal of complementary sequences led to the anticipated length of resulting amplicons to be 427 nt (465 - 17 - 21). The forward reads were truncated at 262 nt, and the reverse reads were truncated at 185 nt, resulting in an ideal overlap of 20 nt. The truncation sites are illustrated in Figure 2. The denoising process of paired-end sequences included dereplication, removal of phiX reads, and identification of chimeric sequences.(3) To account for the lower sequencing quality of the reverse reads, the maximum expected errors were set to 2 for forward reads and 4 for reverse reads. After denoising, a total of 1,446 features were identified. The median frequency of features was 2,434 (ranging from 338 to 3,893).

1.4 Clustering reads into OTUs

Clustering into OTUs is an alternative method for generating feature tables. Initially, the forward and reverse reads were merged.(4) The overlap between the forward and reverse reads was approximately 173 nt (300 + 300 - 427). A maximum of 34 mismatches were permitted in the overlapping sequences to account for potential errors. Subsequently, low-quality reads were filtered out using a minimum Phred quality score of 25.(5) The median number of merged reads was 2,411.5 (ranging from 448 to 4,337 reads). De novo OTU clustering was conducted at a 99% identity threshold after dereplication of the merged reads. Chimeric sequences were identified and removed. The median frequency of features after clustering was 2,091 (ranging from 403 to 3,850).

1.5 Taxonomic classification

The reference sequences from Bacteria and Archaea underwent quality control using REference Sequence annotation and CuRatIon Pipeline.(6) Subsequently, we extracted amplicon-specific sequences based on the complementary sequences in the forward and reverse PCR primers.(7) After dereplication, the Naïve Bayes taxonomy classifier was trained. The classifier's performance under optimal conditions with intentional data leakage is presented in Figure 3-1.

To improve taxonomic classification accuracy, we used the redbiom tool to fetch human fecal samples in the context of "Deblur_2021.09-Illumina-16S-V4-150nt-ac8c0b" from the Qiita database (https://qiita.ucsd.edu).(8, 9) A total of 8,735 samples were obtained as of January 9, 2023. The sequence variants were determined, and the ecologically informed prior class weights were calculated

using q2-clawback.(10) We retrained the Naïve Bayes taxonomy classifier with the prior class weights.(7) The performance of this bespoke taxonomy classifier under optimal conditions with intentional data leakage is presented in Figure 3-2. We then used the bespoke taxonomy classifier to classify features. Features identified as belonging to mitochondria or chloroplasts were discarded. Subsequently, features that were present in only one sample were excluded, followed by the removal of samples with read counts < 1,000.

1.6 Alpha diversities and beta diversities

We aligned the sequences using MAFFT and constructed rooted phylogenetic trees using FastTree with the CAT approximation.(11, 12) The observed number of features, the Shannon's diversity index, and the Faith's phylogenetic diversity were used to estimate alpha diversities across the study groups and between subjects with and without constipation. Statistical significance was determined using the Kruskal-Wallis test by ranks. Unweighted UniFrac distances and weighted UniFrac distances were used to estimate beta diversities.(13, 14) The rarefaction depth was set to 1,000 reads based on the analysis of alpha rarefaction curves and beta rarefaction heatmaps. The structures of the microbial communities were visualized using principal coordinate analysis (PCoA) plots.(15) PERmutational Multivariate ANalysis of Variance (PERMANOVA) was employed to test the hypothesis that within-group distances from each group differ from between-group distances.(16) Q values of pairwise comparisons were calculated using the Benjamini-Hochberg procedure.

1.7 Differential abundance analyses

ANCOM assesses the statistical significance of differences in ratios between a particular feature and the remaining features across the study groups.(17) The analysis yields a W value that represents the number of subhypotheses showing significant differences for the specified feature. Pseudocounts of one were added before the analysis.

ANCOM-BC incorporates a sample-specific offset term to correct for varying sampling fractions across samples, and the estimated absolute abundances are modeled using a linear regression on a log scale.(18) The implementation of ANCOM-BC was carried out in QIIME 2 version 2023.2. Features with a prevalence of < 0.1 were excluded from the analysis.

ALDEx2 identifies features exhibiting greater between-condition to within-condition differences. The process involves converting feature counts into vectors of probabilities through Monte Carlo sampling.(19) These probabilities then undergo centered log-ratio (CLR) transformation. The Wilcoxon rank test is conducted on the elements within the vector.(20) The implementation of ALDEx2 was carried out in QIIME 2 version 2019.7.

MaAsLin 2 offers a comprehensive system that enables the execution of multivariable association testing on microbial data.(21) The "MaAsLin2" R package was used to implement MaAsLin 2.(22) The log-transformed linear model was applied to total-sum-scaled data, effectively controlling false discovery rates. MaAsLin 2 filters out features with a prevalence of < 0.1 by default.

1.8 Microbial correlations

To calculate the Spearman's correlation on CLR-transformed data, pseudocounts of one were added before the transformation.(23) P values for SparCC correlations were determined using FastSpar through 1000 bootstrapping iterations.(24, 25) False discovery rates were controlled using q values calculated by the Benjamini-Hochberg procedure.

SECOM is designed to estimate linear or nonlinear relationships by utilizing distance correlations under the assumption of sparsity.(26, 27) SECOM does not incorporate pseudocounts by default. A prevalence threshold of 0.1 was established to eliminate features with low prevalence while ensuring an adequate number of features for estimating sample-specific biases. Pearson correlation coefficient (PCC) was used in SECOM to measure linear correlation. Since the distance correlation coefficient in SECOM is a measure of general dependency, it is always non-negative. The sparse correlation matrices in SECOM were derived through p value filtering with a default cutoff of 0.005.

1.9 Microbial networks

SCNIC was used to infer the co-occurrence networks and identify modules of co-correlated genera with the shared minimum distance algorithm.(28) To control the network size, edges are defined as correlations with an R value ≥ 0.5 calculated using SparCC.(24) All pairs of features exhibit correlations with an R value ≥ 0.5 in the modules. Network visualization was carried out using Cytoscape.(29)

SPIEC-EASI was employed to infer microbial ecological networks that encompass both positive and negative undirected correlations of conditional independence.(30) Graph estimation was conducted using the Meinshausen-Buhlmann neighborhood selection method. with lambda.min.ratio at 0.1, nlambda at 10, and rep.num at 100.(31) A link in the graphical model indicates a relationship that cannot be explained by any other network. Edges with an absolute weight < 0.1 were removed to control the sizes of networks. The Fruchterman-Reingold algorithm incorporating absolute edge weights was employed to generate layouts.(32)

Figure 1. Sequencing quality of forward and reverse reads.

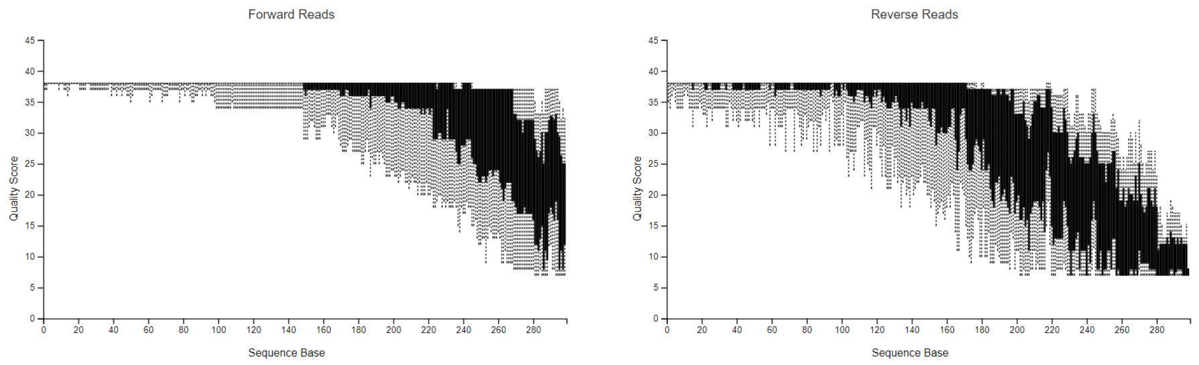

Figure 2. Truncation sites for forward and reverse reads.

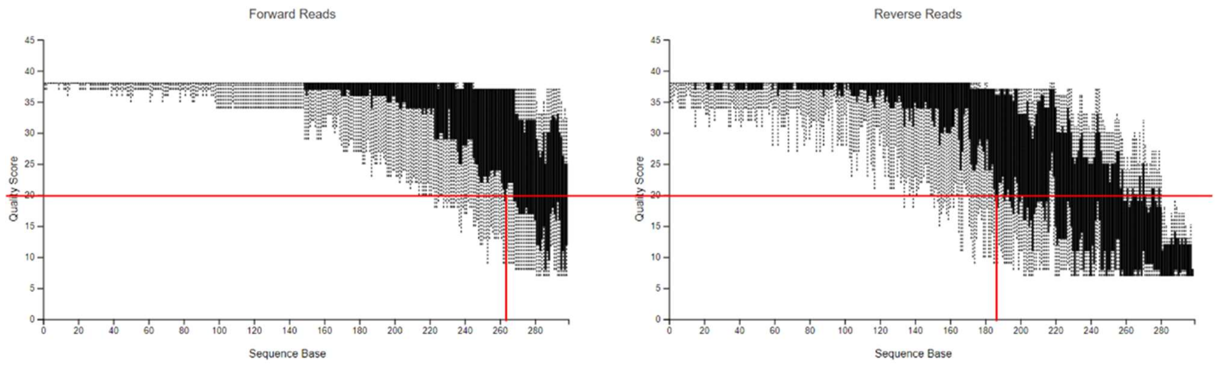

Figure 3-1. The F-measures achieved by the naïve Bayes taxonomy classifier at various taxonomic levels under optimal conditions.

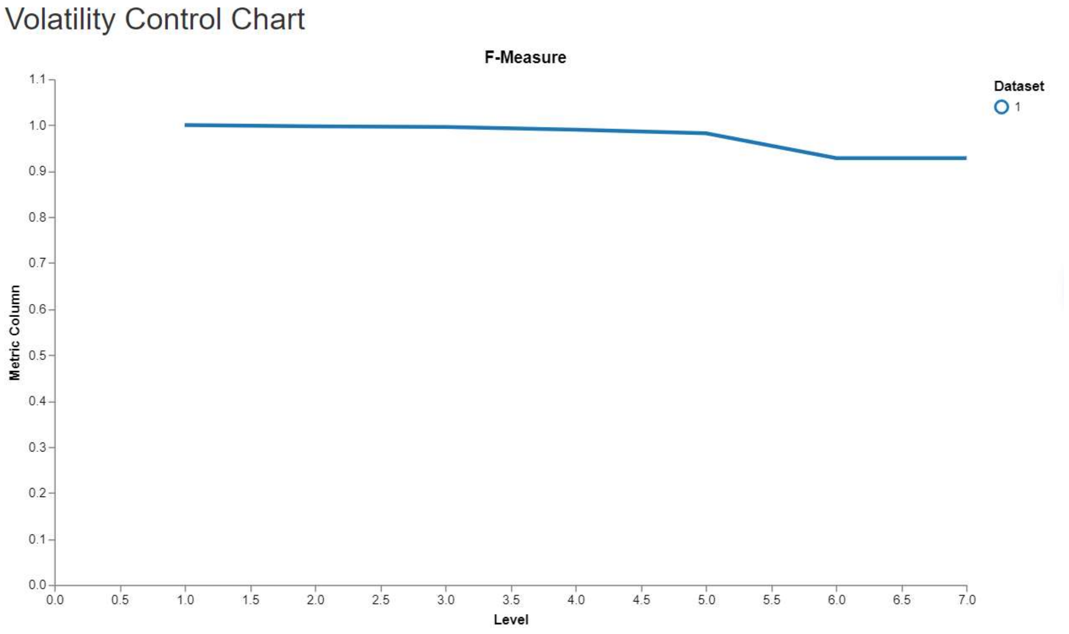

Figure 3-2. The F-measures achieved by the bespoke taxonomy classifier at various taxonomic levels under optimal conditions.

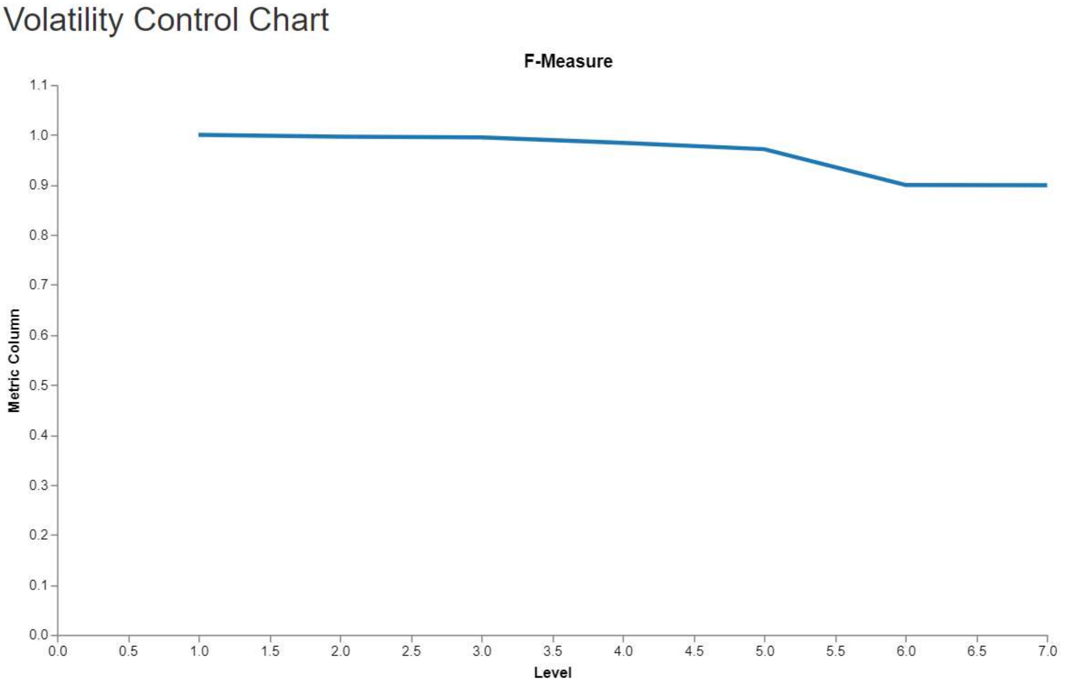

References

1. Bolyen E, Rideout JR, Dillon MR, Bokulich NA, Abnet CC, Al-Ghalith GA, et al. Reproducible, interactive, scalable and extensible microbiome data science using QIIME 2. Nature biotechnology. 2019;37(8):852-7.

2. Hamady M, Walker JJ, Harris JK, Gold NJ, Knight R. Error-correcting barcoded primers for pyrosequencing hundreds of samples in multiplex. Nature methods. 2008;5(3):235-7.

3. Callahan BJ, McMurdie PJ, Rosen MJ, Han AW, Johnson AJ, Holmes SP. DADA2: High-resolution sample inference from Illumina amplicon data. Nature methods. 2016;13(7):581-3.

4. Rognes T, Flouri T, Nichols B, Quince C, Mahé F. VSEARCH: a versatile open source tool for metagenomics. PeerJ. 2016;4:e2584.

5. Bokulich NA, Subramanian S, Faith JJ, Gevers D, Gordon JI, Knight R, et al. Quality-filtering vastly improves diversity estimates from Illumina amplicon sequencing. Nature methods. 2013;10(1):57-9.

6. Robeson MS, II, O'Rourke DR, Kaehler BD, Ziemski M, Dillon MR, Foster JT, et al. RESCRIPt: Reproducible sequence taxonomy reference database management. PLOS Computational Biology. 2021;17(11):e1009581.

7. Bokulich NA, Kaehler BD, Rideout JR, Dillon M, Bolyen E, Knight R, et al. Optimizing taxonomic classification of marker-gene amplicon sequences with QIIME 2's q2-feature-classifier plugin. Microbiome. 2018;6(1):90.

8. McDonald D, Kaehler B, Gonzalez A, DeReus J, Ackermann G, Marotz C, et al. redbiom: a Rapid Sample Discovery and Feature Characterization System. mSystems. 2019;4(4).

9. Gonzalez A, Navas-Molina JA, Kosciolek T, McDonald D, Vázquez-Baeza Y, Ackermann G, et al. Qiita: rapid, web-enabled microbiome meta-analysis. Nature methods. 2018;15(10):796-8.

10. Kaehler BD, Bokulich NA, McDonald D, Knight R, Caporaso JG, Huttley GA. Species abundance information improves sequence taxonomy classification accuracy. Nature Communications. 2019;10(1):4643.

11. Katoh K, Standley DM. MAFFT Multiple Sequence Alignment Software Version 7:

Improvements in Performance and Usability. Molecular Biology and Evolution. 2013;30(4):772-80.

12. Price MN, Dehal PS, Arkin AP. FastTree 2 – Approximately Maximum-Likelihood Trees for Large Alignments. PLOS ONE. 2010;5(3):e9490.

13. Lozupone C, Lladser ME, Knights D, Stombaugh J, Knight R. UniFrac: an effective distance metric for microbial community comparison. The ISME journal. 2011;5(2):169-72.

14. Lozupone CA, Hamady M, Kelley ST, Knight R. Quantitative and qualitative beta diversity measures lead to different insights into factors that structure microbial communities. Applied and environmental microbiology. 2007;73(5):1576-85.

15. Vázquez-Baeza Y, Pirrung M, Gonzalez A, Knight R. EMPeror: a tool for visualizing high-throughput microbial community data. GigaScience. 2013;2(1):16.

16. Anderson MJ. A new method for non-parametric multivariate analysis of variance. Austral Ecology. 2001;26(1):32-46.

17. Mandal S, Van Treuren W, White RA, Eggesbø M, Knight R, Peddada SD. Analysis of composition of microbiomes: a novel method for studying microbial composition. Microbial ecology in health and disease. 2015;26:27663.

18. Lin H, Peddada SD. Analysis of compositions of microbiomes with bias correction. Nature Communications. 2020;11(1):3514.

19. Fernandes AD, Macklaim JM, Linn TG, Reid G, Gloor GB. ANOVA-Like Differential Expression (ALDEx) Analysis for Mixed Population RNA-Seq. PLOS ONE. 2013;8(7):e67019.

20. Fernandes AD, Reid JNS, Macklaim JM, McMurrough TA, Edgell DR, Gloor GB. Unifying the analysis of high-throughput sequencing datasets: characterizing RNA-seq, 16S rRNA gene sequencing and selective growth experiments by compositional data analysis. Microbiome. 2014;2(1):15.

21. Mallick H, Rahnavard A, McIver LJ, Ma S, Zhang Y, Nguyen LH, et al. Multivariable association discovery in population-scale meta-omics studies. PLOS Computational Biology. 2021;17(11):e1009442.

22. Mallick H RA, McIver LJ. MaAsLin 2: Multivariable Association in Population-scale Meta-

omics Studies. R/Bioconductor package. 2020.

23. Kaul A, Mandal S, Davidov O, Peddada SD. Analysis of Microbiome Data in the Presence of Excess Zeros. Frontiers in microbiology. 2017;8:2114.

24. Friedman J, Alm EJ. Inferring Correlation Networks from Genomic Survey Data. PLOS Computational Biology. 2012;8(9):e1002687.

25. Watts SC, Ritchie SC, Inouye M, Holt KE. FastSpar: rapid and scalable correlation estimation for compositional data. Bioinformatics (Oxford, England). 2019;35(6):1064-6.

26. Lin H, Eggesbø M, Peddada SD. Linear and nonlinear correlation estimators unveil undescribed taxa interactions in microbiome data. Nat Commun. 2022;13(1):4946.

27. Székely GJ, Rizzo ML, Bakirov NK. Measuring and Testing Dependence by Correlation of Distances. The Annals of Statistics. 2007;35(6):2769-94.

28. Shaffer M, Thurimella K, Sterrett JD, Lozupone CA. SCNIC: Sparse correlation network investigation for compositional data. Molecular ecology resources. 2023;23(1):312-25.

29. Shannon P, Markiel A, Ozier O, Baliga NS, Wang JT, Ramage D, et al. Cytoscape: a software environment for integrated models of biomolecular interaction networks. Genome research. 2003;13(11):2498-504.

30. Kurtz ZD, Müller CL, Miraldi ER, Littman DR, Blaser MJ, Bonneau RA. Sparse and compositionally robust inference of microbial ecological networks. PLoS Comput Biol. 2015;11(5):e1004226.

31. Meinshausen N, Bühlmann P. High-Dimensional Graphs and Variable Selection with the Lasso. The Annals of Statistics. 2006;34(3):1436-62.

32. Fruchterman TMJ, Reingold EM. Graph drawing by force-directed placement. Software: Practice and Experience. 1991;21(11):1129-64.

2 Supplementary Figures and Tables

2.1 Supplementary Figures

Supplementary Figure 1. PCoA plots illustrating microbial compositions

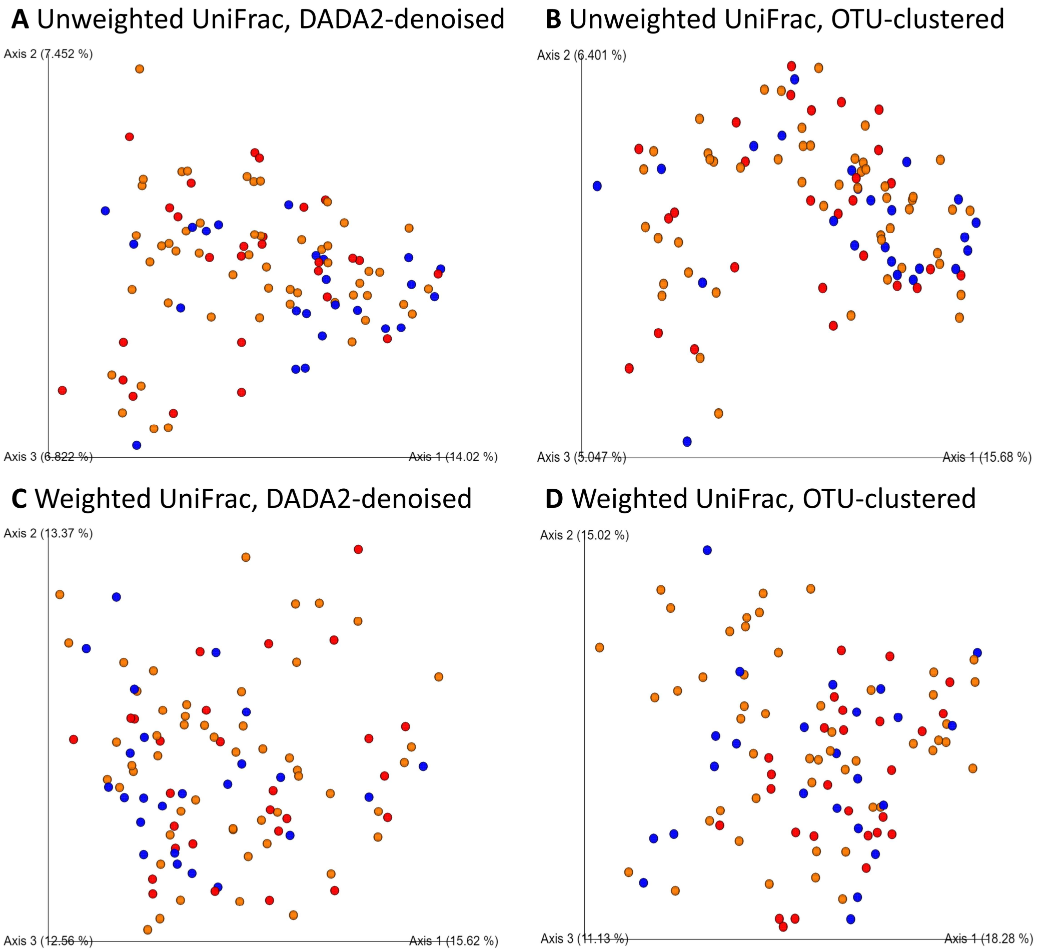

Unweighted UniFrac distances (**A, B**) and weighted UniFrac distances (**C, D**) are displayed. The first and second principal coordinates are shown. MSA samples are denoted by blue dots, PD samples by orange dots, and HC samples by red dots.

Abbreviations: OTU, operational taxonomic unit.

Supplementary Figure 2. Correlation between MSA-associated genera and PD-associated genera (OTU-clustering)

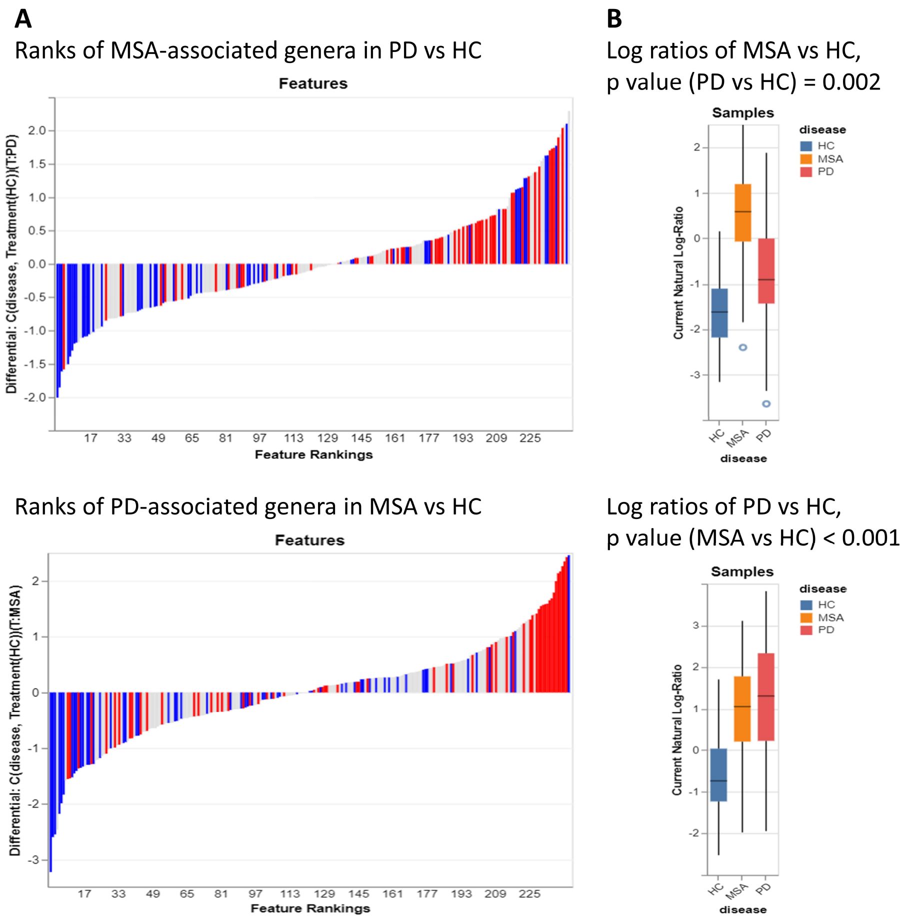

(A) Distribution of the top and bottom 25% of differentially abundant genera (MSA vs HC) in the ranks of relative differentials between PD and HC, and vice versa. The red bars represent the top 25% most abundant genera in MSA (or PD) compared with HC, while the blue bars represent the bottom 25% least abundant genera in MSA (or PD) compared with HC. The ranking of genera was determined by their relative differentials between PD (or MSA) and HC. Songbird was employed to calculate the relative differential of each genus, and Qurro was employed to visualize the ranked relative differentials. OTU-clustering was used in sample preprocessing. (**B)** Log ratios between the top and bottom 25% of differentially abundant genera between MSA and HC and between PD and HC. P values were determined using the Student's t test. Qurro was used to display the distributions of log ratios. OTU-clustering was used in sample preprocessing.

Abbreviations: HC, healthy control; MSA, multiple system atrophy; PD, Parkinson's disease.

Supplementary Figure 3. Predictive accuracy of random forest classifiers in determining disease status

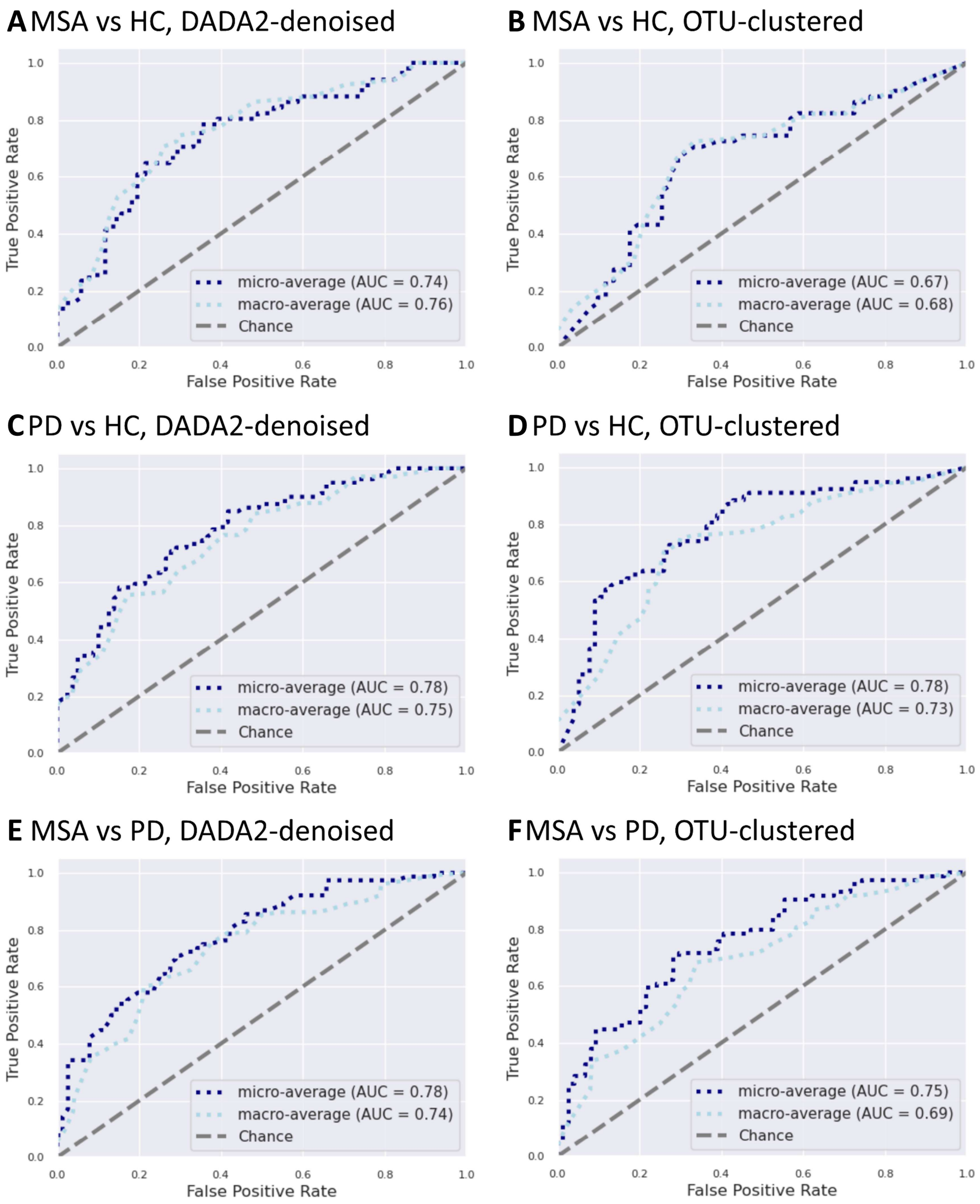

The receiver operating characteristic curve illustrates the relationship between the true positive rate and the false positive rate across various thresholds of random forest classifiers. (**A-D)** A patient predicted to have the disease vs a HC predicted to have the disease. (**E, F)** A MSA patient predicted to have MSA vs a MSA patient predicted to have PD. The AUC quantifies the performance of the classifiers. ”Micro-averaging AUC” computes metrics by averaging across individual samples,

while ”macro-averaging AUC” gives equal weight to the classification of each sample. Only “micro-averaging AUCs” were addressed in the manuscript.

Abbreviations: AUC, area under the curve; HC, healthy control; MSA, multiple system atrophy; OTU, operational taxonomic unit; PD, Parkinson’s disease.

Supplementary Figure 4-1. Microbial correlation matrices in MSA (OTU-clustering)

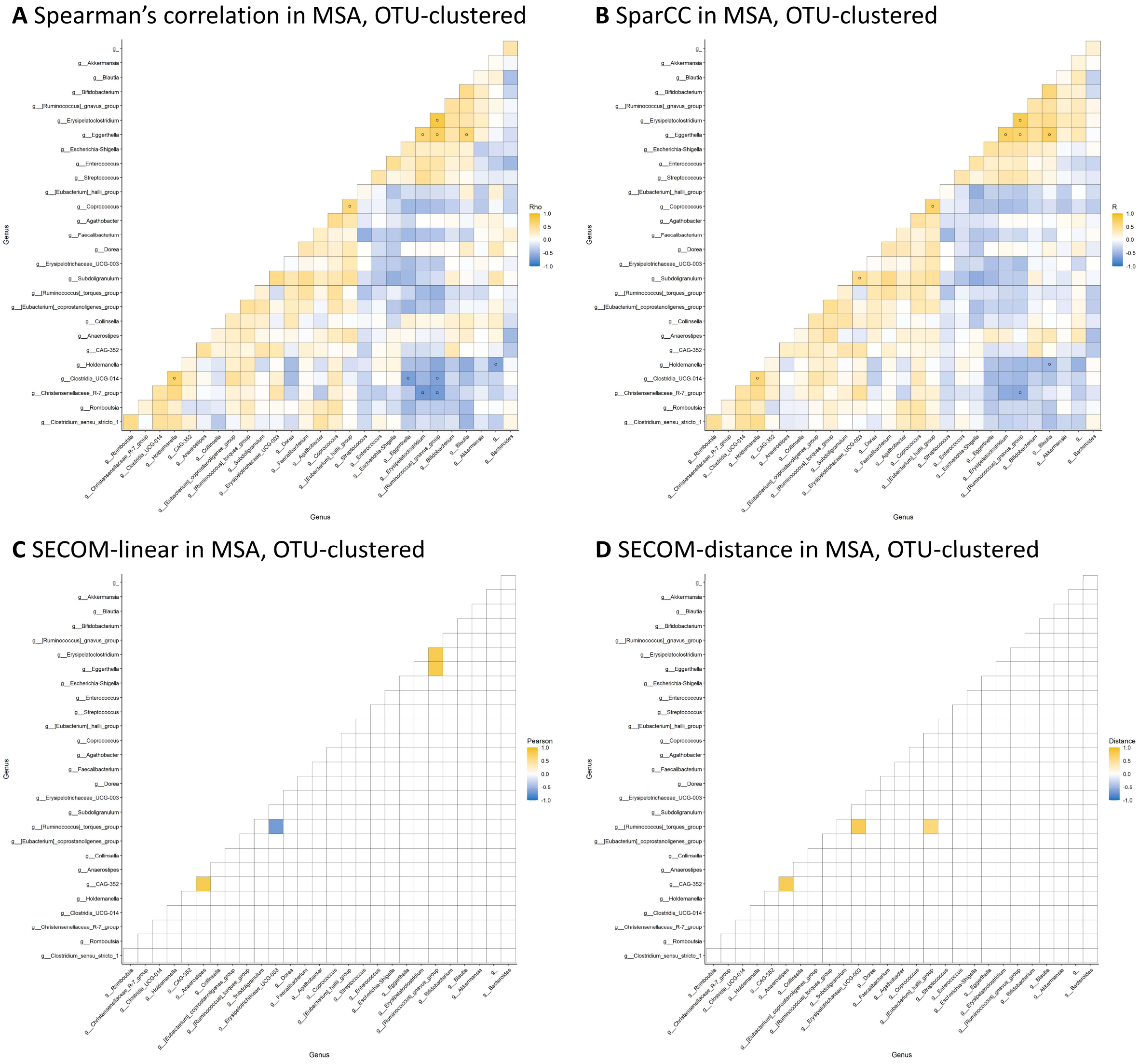

Four methods were employed to calculate the microbial correlation matrices, including the Spearman's correlation on CLR-transformed data (**A**), SparCC (**B**), SECOM using linear correlation (**C**), and distance correlation (**D**). Only genera with a prevalence ≥ 50% and an average abundance ≥ 20 in each study group were considered for analyses. The correlation coefficient, which ranges from −1 to 1, was represented using a color gradient from blue to yellow. Statistical significance is denoted by small circles following correction for multiple comparisons. The order of the genera was identical across all four matrices. OTU-clustering was used in sample preprocessing.

Abbreviations: MSA, multiple system atrophy; OTU, operational taxonomic unit; SECOM, Sparse Estimation of Correlations among Microbiomes.

Supplementary Figure 4-2. Microbial correlation matrices in PD (DADA2-denoising)

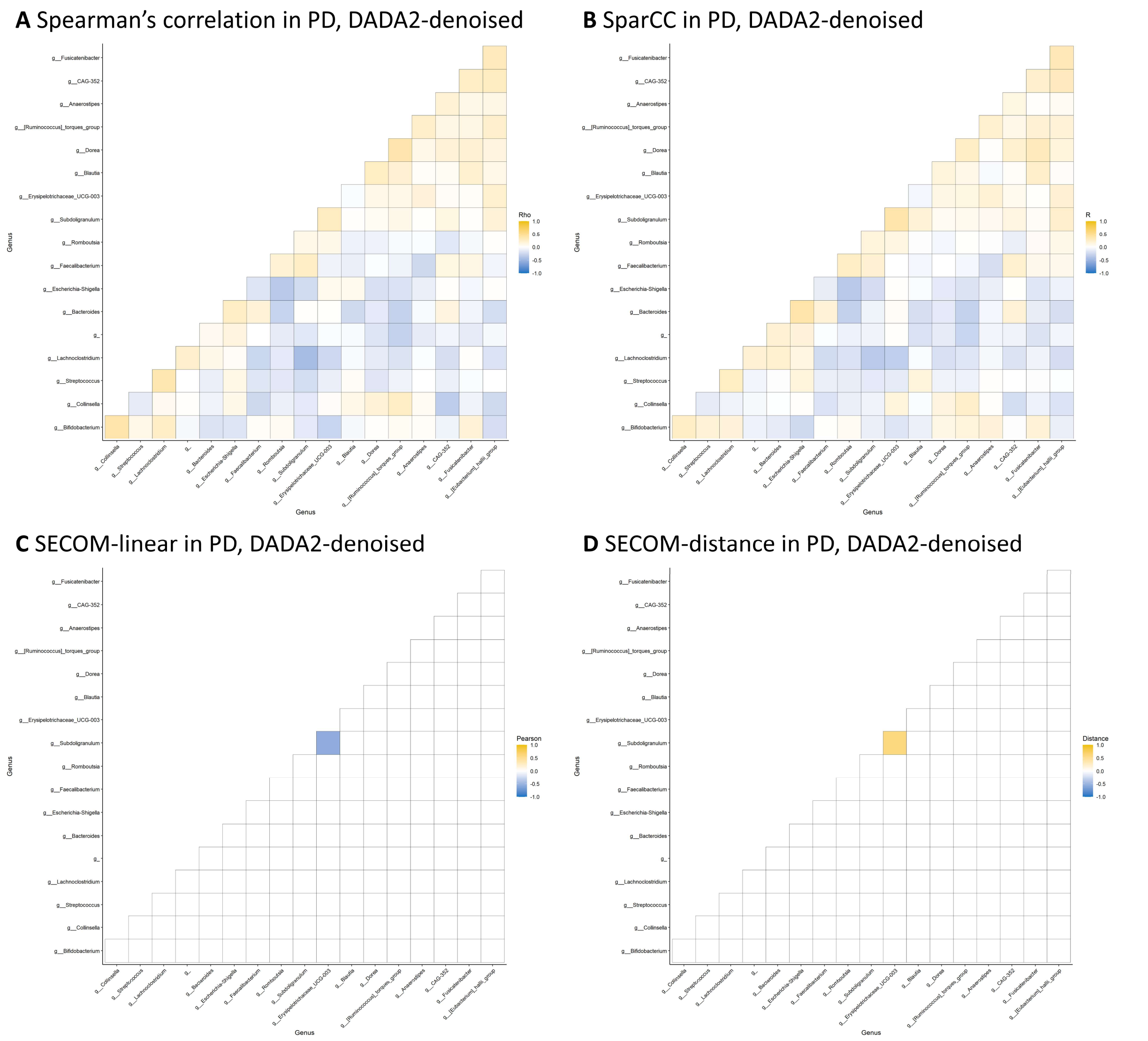

Four methods were employed to calculate the microbial correlation matrices, including the Spearman's correlation on CLR-transformed data (**A**), SparCC (**B**), SECOM using linear correlation (**C**), and distance correlation (**D**). Only genera with a prevalence ≥ 50% and an average abundance ≥ 20 in each study group were considered for analyses. The correlation coefficient, which ranges from −1 to 1, was represented using a color gradient from blue to yellow. Statistical significance is denoted by small circles following correction for multiple comparisons. The order of the genera was identical across all four matrices. DADA2-denoising was used in sample preprocessing.

Abbreviations: PD, Parkinson's disease; SECOM, Sparse Estimation of Correlations among Microbiomes.

Supplementary Figure 4-3. Microbial correlation matrices in PD (OTU-clustering)

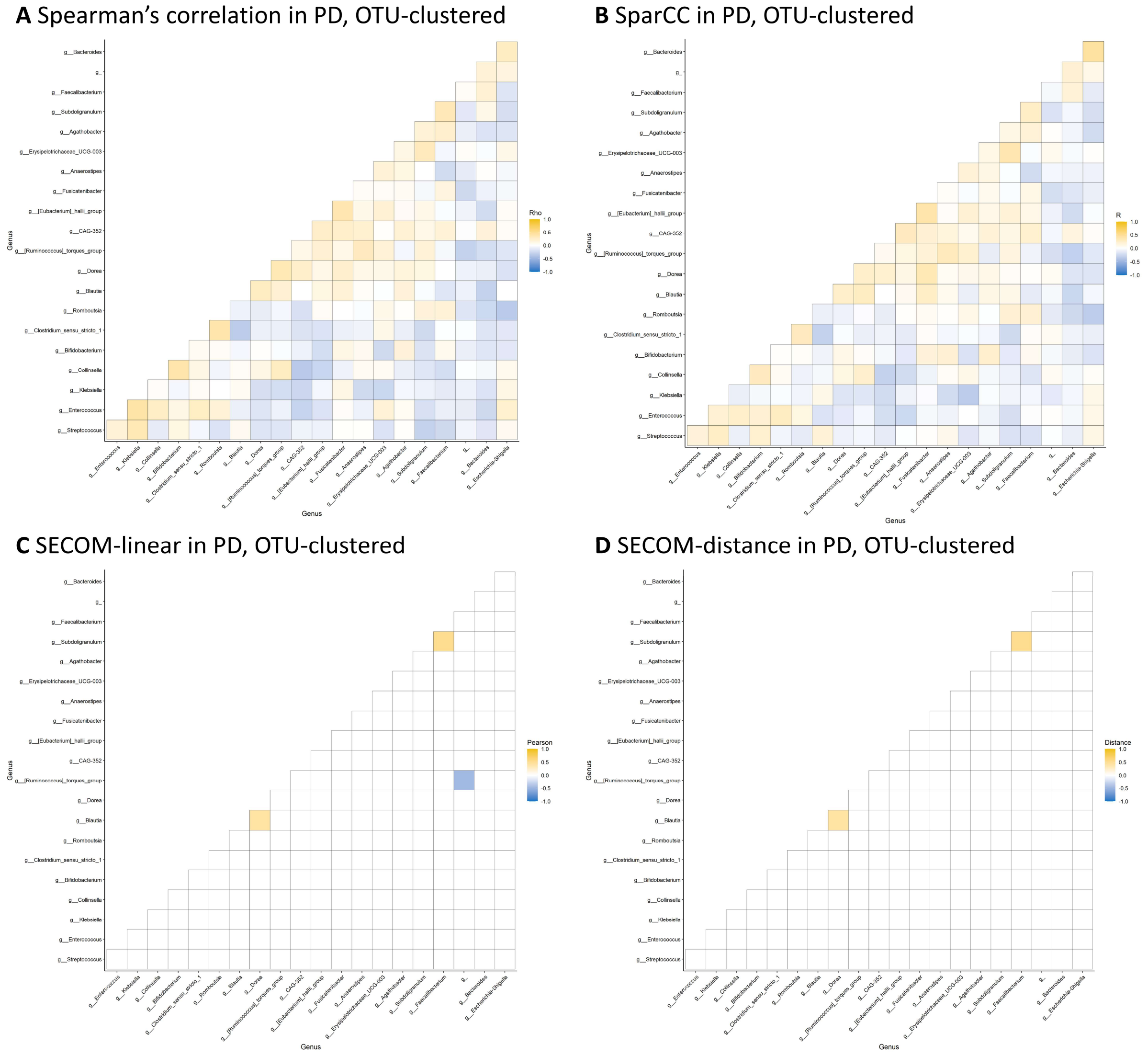

Four methods were employed to calculate the microbial correlation matrices, including the Spearman's correlation on CLR-transformed data (**A**), SparCC (**B**), SECOM using linear correlation (**C**), and distance correlation (**D**). Only genera with a prevalence ≥ 50% and an average abundance ≥ 20 in each study group were considered for analyses. The correlation coefficient, which ranges from −1 to 1, was represented using a color gradient from blue to yellow. Statistical significance is denoted by small circles following correction for multiple comparisons. The order of the genera was identical across all four matrices. OTU-clustering was used in sample preprocessing.

Abbreviations: OTU, operational taxonomic unit; PD, Parkinson's disease; SECOM, Sparse Estimation of Correlations among Microbiomes.

Supplementary Figure 4-4. Microbial correlation matrices in HC (DADA2-denoising)

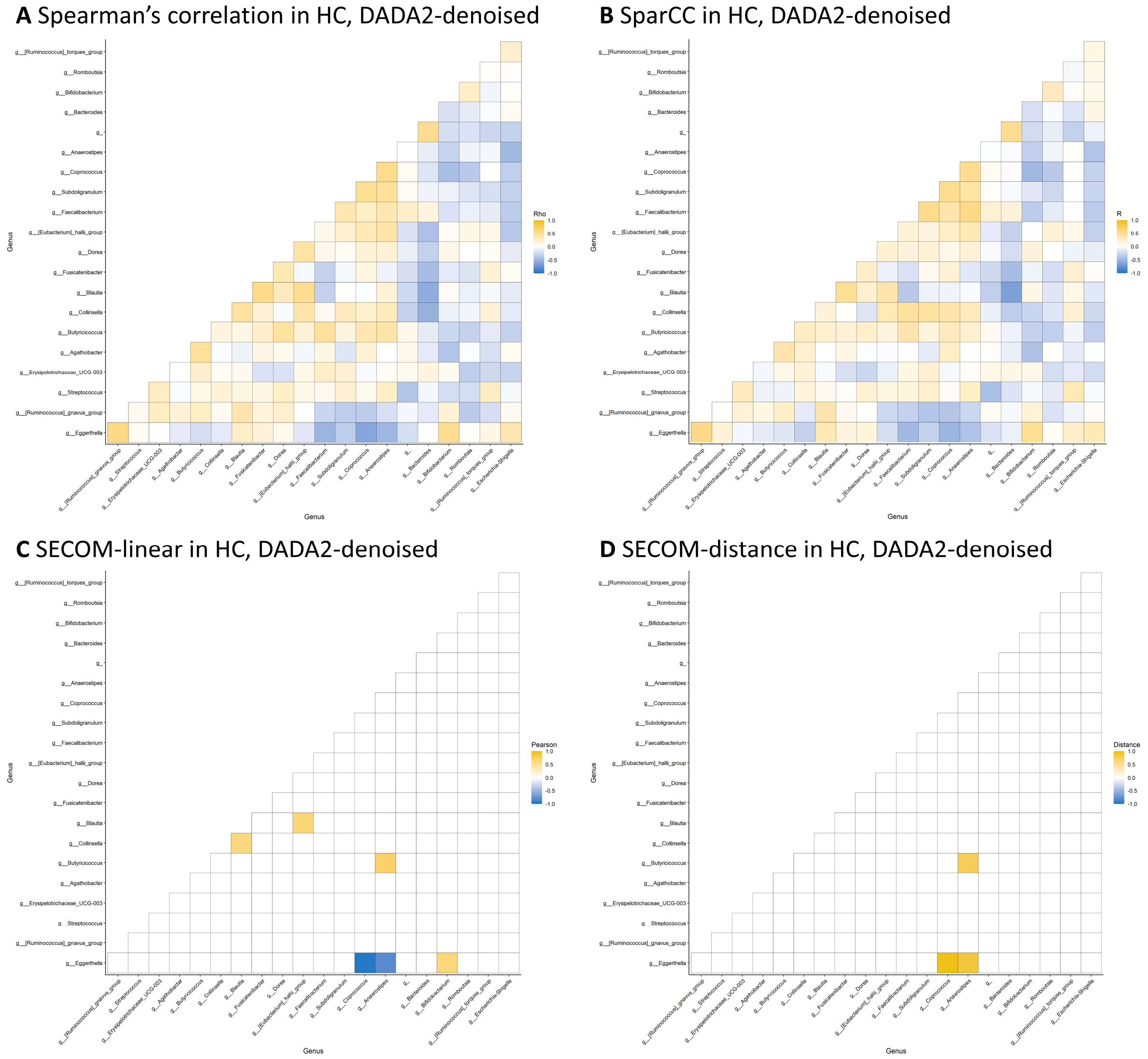

Four methods were employed to calculate the microbial correlation matrices, including the Spearman's correlation on CLR-transformed data (**A**), SparCC (**B**), SECOM using linear correlation (**C**), and distance correlation (**D**). Only genera with a prevalence ≥ 50% and an average abundance ≥ 20 in each study group were considered for analyses. The correlation coefficient, which ranges from −1 to 1, was represented using a color gradient from blue to yellow. Statistical significance is denoted by small circles following correction for multiple comparisons. The order of the genera was identical across all four matrices. DADA2-denoising was used in sample preprocessing.

Abbreviations: HC, healthy control; SECOM, Sparse Estimation of Correlations among Microbiomes.

Supplementary Figure 4-5. Microbial correlation matrices in HC (OTU-clustering)

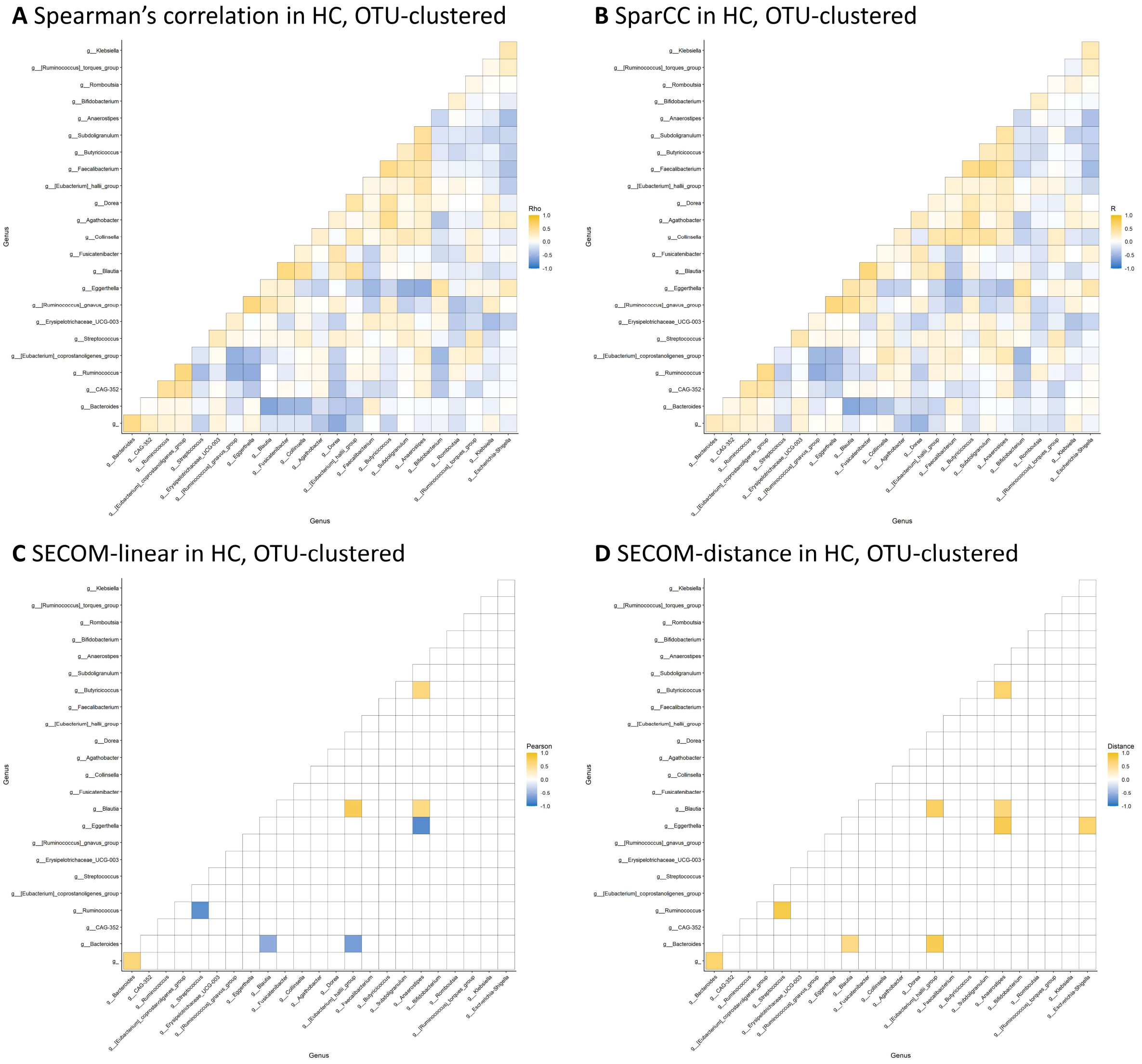

Four methods were employed to calculate the microbial correlation matrices, including the Spearman's correlation on CLR-transformed data (**A**), SparCC (**B**), SECOM using linear correlation (**C**), and distance correlation (**D**). Only genera with a prevalence ≥ 50% and an average abundance ≥ 20 in each study group were considered for analyses. The correlation coefficient, which ranges from −1 to 1, was represented using a color gradient from blue to yellow. Statistical significance is denoted by small circles following correction for multiple comparisons. The order of the genera was identical across all four matrices. OTU-clustering was used in sample preprocessing.

Abbreviations: HC, healthy control; OTU, operational taxonomic unit; SECOM, Sparse Estimation of Correlations among Microbiomes.

Supplementary Figure 5-1. Microbial networks in PD

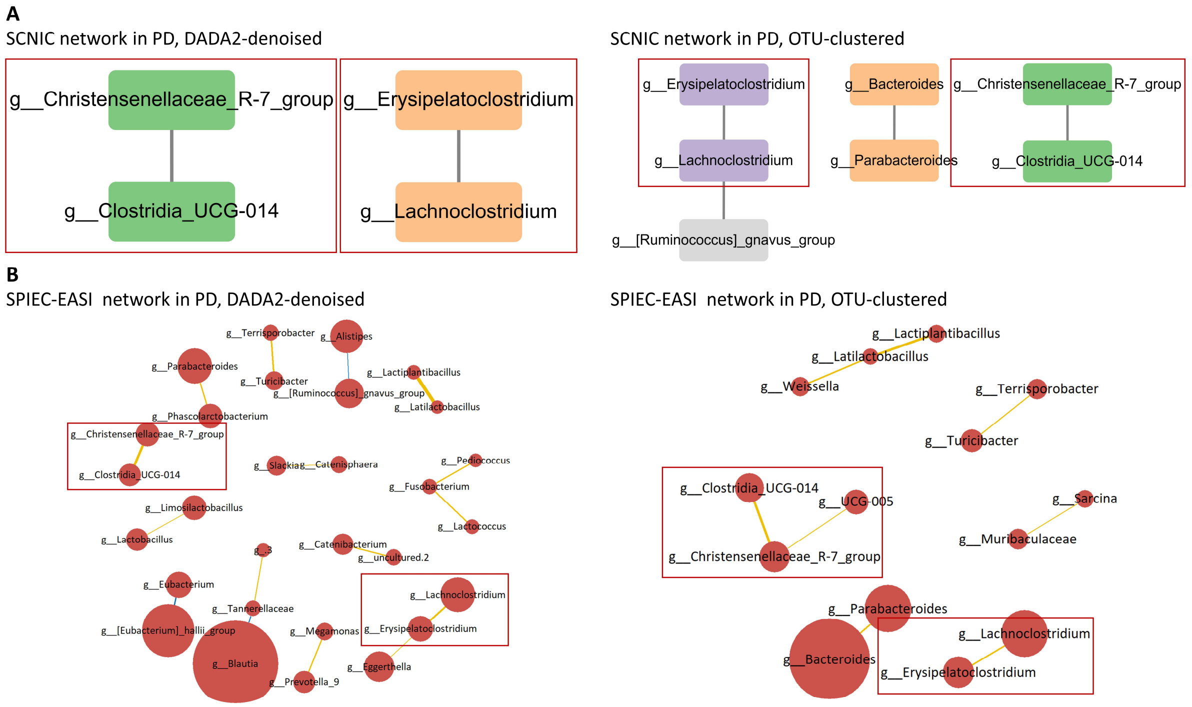

(A) In SCNIC networks, edges are defined as correlations with an R value ≥ 0.5. The color of the nodes indicates their membership in a particular module. Visualization of the SCNIC networks was achieved using Cytoscape. The interbacterial interactions consistently identified using both SCNIC and SPIEC-EASI are highlighted. (**B**) In SPIEC-EASI networks, the node size is scaled according to the mean of CLR-transformed data, and the edge width is proportional to the absolute weight of the connection. Positive correlations are denoted by orange edges, while negative correlations are denoted by blue edges. Only edges with an absolute weight ≥ 0.1 are shown. The interbacterial interactions consistently identified using both SCNIC and SPIEC-EASI are highlighted.

Abbreviations: OTU, operational taxonomic unit; PD, Parkinson's disease; SCNIC, Sparse Cooccurrence Network Investigation for Compositional data; SPIEC-EASI, SParse InversE Covariance Estimation for Ecological Association Inference.

Supplementary Figure 5-2. Microbial networks in HC

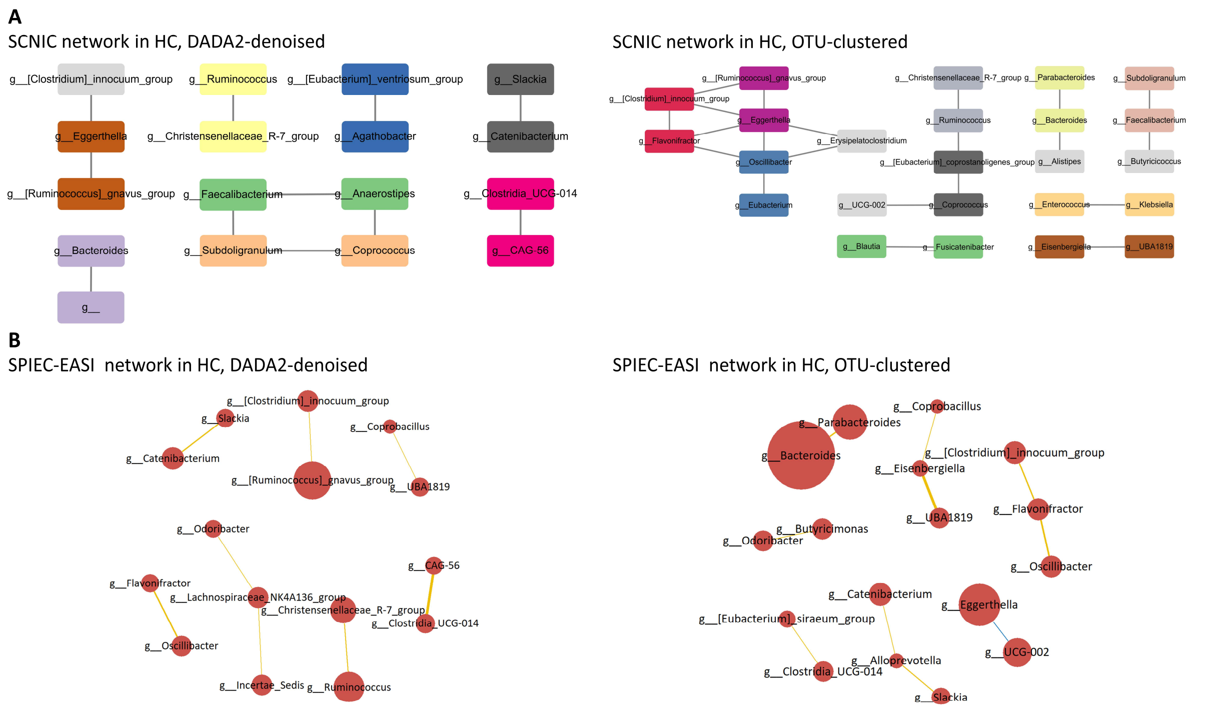

(A) In SCNIC networks, edges are defined as correlations with an R value ≥ 0.5. The color of the nodes indicates their membership in a particular module. Visualization of the SCNIC networks was achieved using Cytoscape. (**B)** In SPIEC-EASI networks, the node size is scaled according to the mean of CLR-transformed data, and the edge width is proportional to the absolute weight of the connection. Positive correlations are denoted by orange edges, while negative correlations are denoted by blue edges. Only edges with an absolute weight ≥ 0.1 are shown.

Abbreviations: HC, healthy control; OTU, operational taxonomic unit; SCNIC, Sparse Cooccurrence Network Investigation for Compositional data; SPIEC-EASI, SParse InversE Covariance Estimation for Ecological Association Inference.

Supplementary Figure 6-1. PCoA plots illustrating microbial compositions associated with constipation status

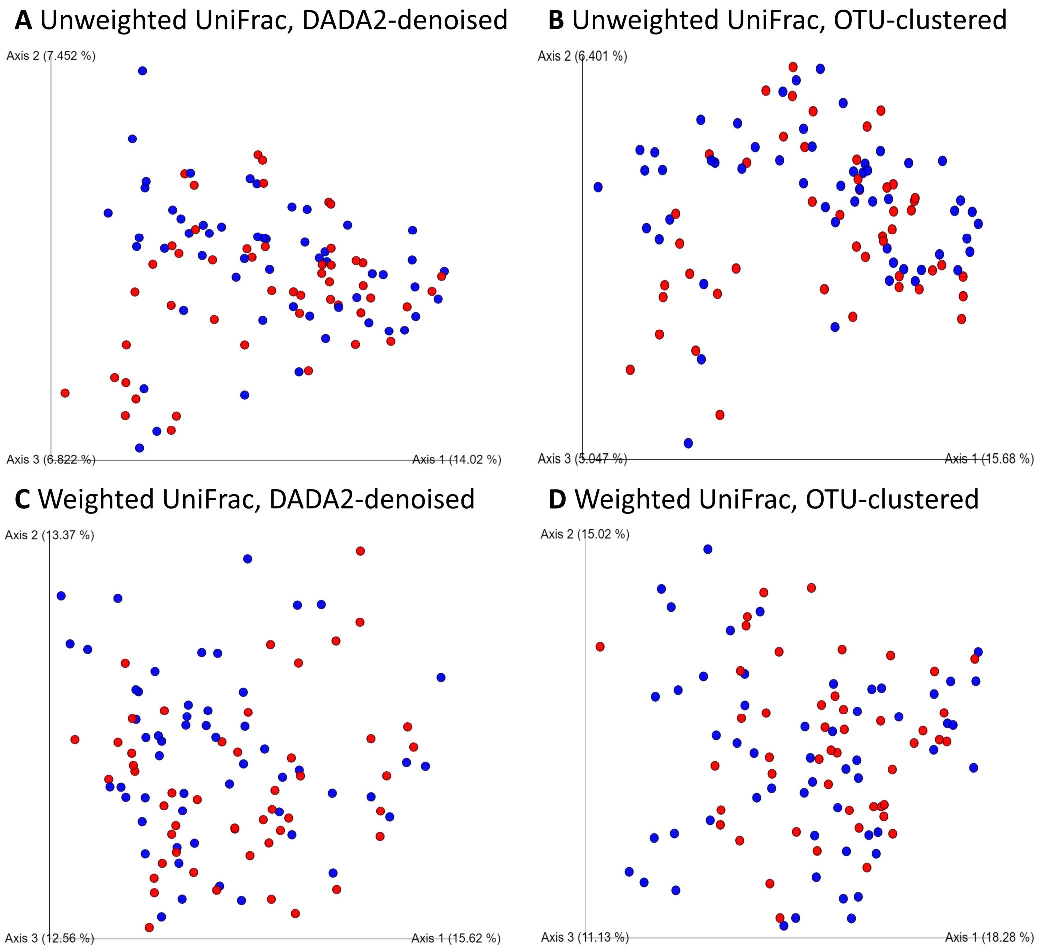

The first and second principal coordinates are presented. Subjects with constipation are denoted by blue dots, and subjects without constipation are denoted by red dots.

Abbreviations: OTU, operational taxonomic unit.

Supplementary Figure 6-2. PCoA plots illustrating microbial compositions associated with constipation status in HC

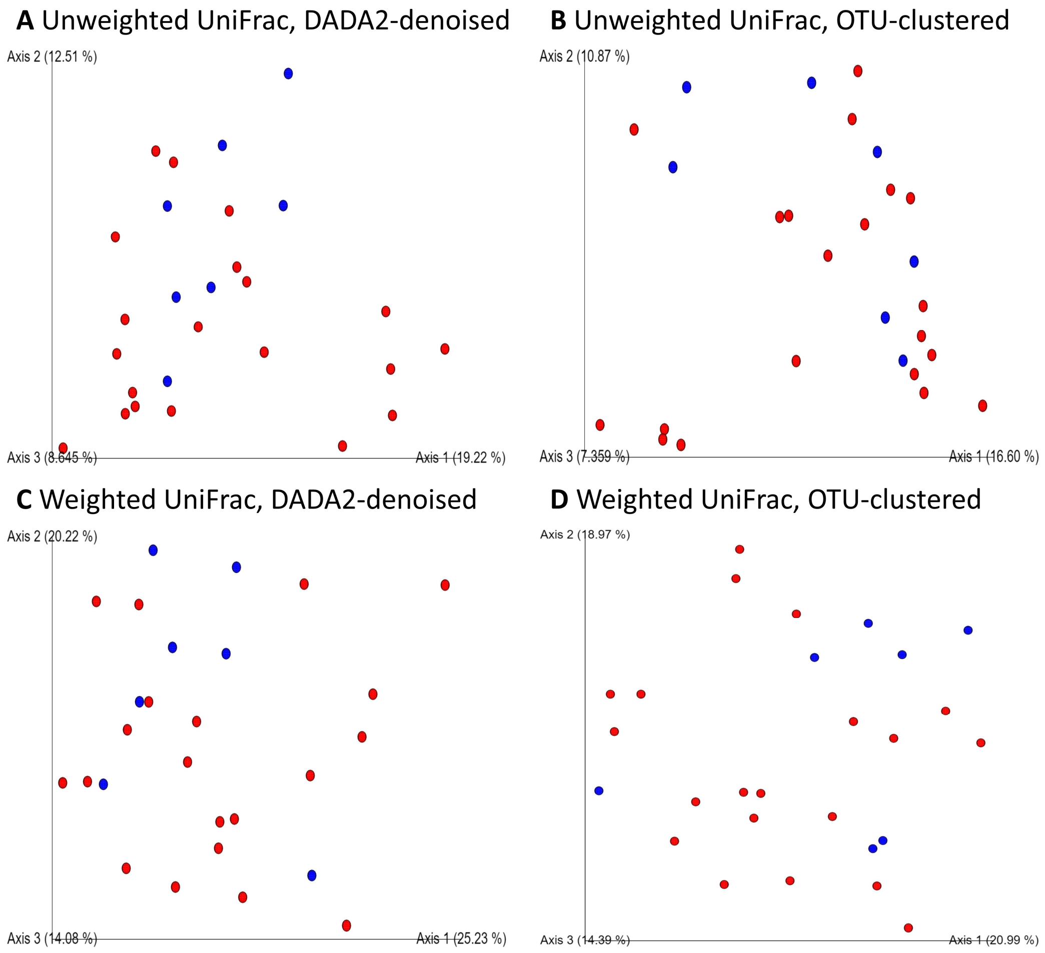

The first and second principal coordinates are presented. Subjects with constipation are denoted by blue dots, and subjects without constipation are denoted by red dots.

Abbreviations: OTU, operational taxonomic unit.

2.2 Supplementary Tables

Supplementary Table 1. Clinicodemographic characteristics associated with constipation status

Characteristics	MSA			PD			HC		
	Constipation	No constipation	p value	Constipation	No constipation	p value	Constipation	No constipation	p value
No.	19	5		28	22		7	20	
Sex			1.000			0.407			1.000
Male	10 (52.6%)	3 (60.0%)		13 (46.4%)	13 (59.1%)		2 (28.6%)	5 (25.0%)	
Female	9 (47.4%)	2 (40.0%)		15 (53.6%)	9 (40.9%)		5(71.4%)	15 (75.0%)	
Age (years)	60.7 ± 8.5	66.8 ± 6.6	0.156	72.6 ± 9.5	75.2 ± 6.9	0.289	70.9 ± 13.6	65.8 ± 7.1	0.376
Hypertension			0.568			0.389			0.662
Yes	4 (21.1%)	2 (40.0%)		9 (32.1%)	10 (45.5%)		5 (71.4%)	11 (55.0%)	
No	15 (79.0%)	3 (60.0%)		19 (67.9%)	12 (54.6%)		2 (28.6%)	9 (45.0%)	
Diabetes mellitus			1.000			0.157			1.000
Yes	2 (10.5%)	0 (0.0%)		3 (10.7%)	6 (27.3%)		1 (14.3%)	2 (10.0%)	
No	17 (89.5%)	5 (100.0%)		25 (89.3%)	16 (72.7%)		6 (85.7%)	18 (90.0%)	
Hyperlipidemia			0.568			0.108			0.077
Yes	4 (21.1%)	2 (40.0%)		10 (35.7%)	3 (13.6%)		1 (14.3%)	12 (60.0%)	
No	15 (79.0%)	3 (60.0%)		18 (64.3%)	19 (86.4%)		6 (85.7%)	8 (40.0%)	
Medications for constipation			0.037			<.001			0.012
Yes	12 (63.2%)	0 (0.0%)		17 (60.7%)	0 (0.0%)		3 (42.9%)	0 (0.0%)	
No	7 (36.8%)	5 (100.0%)		11 (39.3%)	22 (100.0%)		4 (57.1%)	20 (100.0%)	
Usage of probiotics			1.000			1.000			1.000
Yes	10 (52.6%)	2 (40.0%)		14 (50.0%)	11 (50.0%)		0 (0.0%)	2 (10.0%)	

No	9 (47.4%)	3 (60.0%)		14 (50.0%)	11 (50.0%)		7 (100.0%)	18 (90.0%)	
Daily vegetables			0.289			1.000			0.068
Yes	14 (73.7%)	2 (40.0%)		19 (67.9%)	15 (68.2%)		7 (100.0%)	12 (60.0%)	
No	5 (26.3%)	3 (60.0%)		9 (32.1%)	7 (31.8%)		0 (0.0%)	8 (40.0%)	
Age of onset (years)	57.9 ± 7.9	65.4 ± 6.2	0.064	67.8 ± 9.2	72.0 ± 7.3	0.097	---	---	---
Disease duration (years)	2.1 ± 1.4	1.4 ± 0.5	0.274	4.8 ± 5.8	3.1 ± 2.1	0.173	---	---	---
UPDRS III	---	---	---	18.5 ± 13.5	10.2 ± 6.7	0.011	---	---	---

The categorical variables are presented as count (percentage). The numeric variables are presented as mean ± standard deviation. The Fisher's exact test was used to compare categorical variables between subjects with and without constipation. The Student's t test was used to compare age, age of onset, and disease duration between subjects with and without constipation.

Abbreviations: HC, healthy control; MSA, multiple system atrophy; PD, Parkinson's disease; UPDRS, Unified Parkinson's Disease Rating Scale.

Supplementary Table 2. Beta diversities

	MSA vs HC			PD vs HC			MSA vs PD		
	Pseudo-F	p value	q value	Pseudo-F	p value	q value	Pseudo-F	p value	q value
Unweighted UniFrac distance	DADA2-denoising								
	2.44	0.003	0.009	1.97	0.010	0.015	1.54	0.049	0.049
	OTU-clustering								
	1.92	0.014	0.042	1.00	0.445	0.445	1.32	0.101	0.152
Weighted UniFrac distance	DADA2-denoising								
	3.02	0.004	0.012	1.92	0.042	0.063	1.75	0.065	0.065
	OTU-clustering								
	2.84	0.007	0.015	2.43	0.010	0.015	2.10	0.041	0.041

The pseudo-F and p values were estimated using PERmutational Multivariate ANalysis of VAriance. The q values for pairwise comparisons were calculated using the Benjamini-Hochberg procedure.

Abbreviations: HC, healthy control; MSA, multiple system atrophy; OTU, operational taxonomic unit; PD, Parkinson’s disease.

Supplementary Table 3. Multivariable differential analyses of significant genera identified in univariate analyses

	Univariate analysis		Model 1		Model 2	
	LFC/Coefficient	q value	LFC/Coefficient	p value	LFC/Coefficient	p value
ANCOM-BC						
Fusicatenibacter, MSA vs HC						
DADA2-denoising	-2.08	<.001	-2.02	0.001	-1.90	0.002
OTU-clustering	-1.93	<.001	-1.86	<.001	-1.69	<.001
Butyricicoccus, PD vs HC						
DADA2-denoising	-1.29	0.019	-1.09	0.004	-1.14	0.008
OTU-clustering	-1.43	<.001	-1.38	<.001	-1.24	<.001
MaAsLin 2						
Fusicatenibacter, MSA vs HC						
DADA2-denoising	-2.26	0.013	-2.23	0.001	-2.23	0.006
OTU-clustering	-2.72	0.013	-2.54	0.002	-2.42	0.009
Butyricicoccus, PD vs HC						
DADA2-denoising	-1.76	0.008	-1.63	<.001	-1.64	0.002
OTU-clustering	-2.01	0.008	-1.91	<.001	-1.93	0.002

Model 1 adjusted for constipation status. Model 2 adjusted for age, sex, hypertension, constipation status, medications for constipation, and usage of probiotics. The LFC quantifies the effect of study group on the bias-corrected absolute abundance of a particular genus in ANCOM-BC. The "coefficient" refers to the effect size in the linear model, representing the difference between categorical variables in MaAsLin 2. A positive LFC/"coefficient" suggests that the genus is more abundant in the first study group. The q values in ANCOM-BC were determined using the Holm-Bonferroni method, while the q values in MaAsLin 2 were determined using the Benjamini-Hochberg procedure.

Abbreviations: ANCOM-BC, ANalysis of COmposition of Microbiomes with Bias Correction; HC, healthy control; LFC, log fold change; MaAsLin 2,

Microbiome Multivariable Associations with Linear Models 2; MSA, multiple system atrophy; OTU, operational taxonomic unit; PD, Parkinson's disease.

Supplementary Table 4. Significant genera identified in pairwise multivariable ANCOM-BC comparisons

	MSA vs HC			PD vs HC			MSA vs PD		
	Genus	LFC	q value	Genus	LFC	q value	Genus	LFC	q value
Model 1	DADA2-denoising								
	Agathobacter	-1.95	0.038	*Limosilactobacillus*	1.00	0.016	*Limosilactobacillus*	-0.96	0.022
	Phascolarctobacterium	-1.47	0.012	---	---	---	---	---	---
	OTU-clustering								
	Fusicatenibacter	-1.86	0.027	*Butyricicoccus*	-1.38	<.001	---	---	---
	Butyricicoccus	-1.63	0.004	*Bifidobacterium*	-1.35	0.044	---	---	---
	Phascolarctobacterium	-1.50	0.009	---	---	---	---	---	---
	Eubacterium ventriosum group	-1.35	0.041	---	---	---	---	---	---
	Eubacterium brachy group	0.89	0.013	---	---	---	---	---	---
Model 2	DADA2-denoising								
	Agathobacter	-2.28	0.004	***Agathobacter***	-2.04	<.001	---	---	---
	Butyricicoccus	-2.20	<.001	---	---	---	---	---	---
	Ruminococcaceae incertae sedis	1.16	0.017	---	---	---	---	---	---
	Intestinibacter	1.91	0.044	---	---	---	---	---	---
	OTU-clustering								
	Butyricicoccus	-1.89	<.001	***Agathobacter***	-2.03	<.001	---	---	---
	Intestinibacter	1.83	0.014	*Butyricicoccus*	-1.24	0.040	---	---	---
	---	---	---	*Solobacterium*	0.30	0.015	---	---	---
	---	---	---	*Atopobium*	0.37	0.017	---	---	---
	---	---	---	*Faecalicoccus*	0.51	0.002	---	---	---
	---	---	---	*Catenisphaera*	0.94	0.040	---	---	---

Model 1 adjusted for constipation status. Model 2 adjusted for age, sex, hypertension, constipation status, medications for constipation, and usage of probiotics. The LFC quantifies the effect of study group on the bias-corrected absolute abundance of a particular genus. A positive LFC suggests that the

genus is more abundant in the first study group. The q values were determined using the Holm-Bonferroni method. The genera highlighted in bold were identified using both DADA2-denoising and OTU-clustering.

Abbreviations: HC, healthy control; LFC, log fold change; MSA, multiple system atrophy; OTU, operational taxonomic unit; PD, Parkinson's disease.

Supplementary Table 5. Significant genera identified in pairwise multivariable MaAsLin 2 comparisons

	MSA vs HC			PD vs HC			MSA vs PD		
	Genus	Coefficient	q value	Genus	Coefficient	q value	Genus	Coefficient	q value
Model 1	DADA2-denoising								
	---	---	---	***Butyricicoccus***	-1.63	0.038	---	---	---
	OTU-clustering								
	---	---	---	***Butyricicoccus***	-1.91	0.035	---	---	---
Model 2	DADA2-denoising								
	Butyricicoccus	-2.38	0.035	---	---	---	---	---	---
	Intestinibacter	1.97	0.035	---	---	---	---	---	---
	OTU-clustering								
	Butyricicoccus	-3.20	0.036	*Agathobacter*	-3.45	0.042	---	---	---
	Intestinibacter	3.33	0.036	---	---	---	---	---	---

Model 1 adjusted for constipation status. Model 2 adjusted for age, sex, hypertension, constipation status, medications for constipation, and usage of probiotics. The "coefficient" refers to the effect size in the linear model, representing the difference between categorical variables. A positive "coefficient" suggests that the genus is more abundant in the first study group. The q values were determined using the Benjamini-Hochberg procedure. The genera highlighted in bold were identified using both DADA2-denoising and OTU-clustering.

Abbreviations: HC, healthy control; MSA, multiple system atrophy; OTU, operational taxonomic unit; PD, Parkinson's disease.

Supplementary Table 6. Confusion matrices of predictive performance of random forest classifiers

MSA-HC classifier				PD-HC classifier				MSA-PD classifier			
	Predicted to have MSA	Predicted to be HC	AUC		Predicted to have PD	Predicted to be HC	AUC		Predicted to have MSA	Predicted to be PD	AUC
DADA2-denoising											
MSA	58.3%	41.7%	0.74	PD	92.3%	7.7%	0.78	MSA	20.8%	79.2%	0.78
HC	18.5%	81.5%		HC	70.4%	29.6%		PD	5.8%	94.2%	
OTU-clustering											
MSA	62.5%	37.5%	0.67	PD	90.0%	10.0%	0.78	MSA	25.0%	75.0%	0.75
HC	25.9%	74.1%		HC	59.3%	40.7%		PD	8.0%	92.0%	

The percentages were calculated by dividing the number of subjects predicted to have a particular disease status by the total number of actual subjects.

Abbreviations: AUC, area under the curve; HC, health control; MSA, multiple system atrophy; OTU, operational taxonomic unit; PD, Parkinson’s disease.

Supplementary Table 7. Significant interbacterial interactions in network analyses

MSA	PD	HC
SCNIC		
Blautia * *Bifidobacterium*	***Christensenellaceae R-7* group * *Clostridia UCG-014***	*Clostridium innocuum* group * *Eggerthella*
Blautia * *Eggerthella*	***Erysipelatoclostridium* * *Lachnoclostridium***	*Eggerthella* * *Ruminococcus gnavus* group
Blautia * *Sellimonas*	---	*Ruminococcus* * *Christensenellaceae R-7* group
Eggerthella * *Sellimonas*	---	*Faecalibacterium* * *Subdoligranulum*
Eggerthella * *Ruminococcus gnavus* group	---	---
Eggerthella * *Erysipelatoclostridium*	---	---
Sellimonas * *Ruminococcus gnavus* group	---	---
Sellimonas * *Erysipelatoclostridium*	---	---
Ruminococcus gnavus* group * *Erysipelatoclostridium	---	---
Coprococcus * *Eubacterium hallii* group	---	---
Holdemanella * *Ruminococcaceae incertae sedis*	---	---
Alistipes * *Ruminococcus*	---	---
Bacteroides * *Parabacteroides*	---	---
SPIEC-EASI		
Ruminococcus gnavus* group * *Erysipelatoclostridium	*Lactiplantibacillus* * *Latilactobacilllus*	*Slackia* * *Catenibacterium*
Pediococcus * *Lapidilactobacillus*	*Terrisporobacter* * *Turicibacter*	*Coprobacillus* * *Ruminococcaceae UBA1819*
---	***Christensenellaceae R-7* group * *Clostridia UCG-014***	*Flavonifractor* * *Oscillibacter*
---	***Erysipelatoclostridium* * *Lachnoclostridium***	---

The interbacterial interactions listed above were identified using both DADA2-denoising and OTU-clustering. The interbacterial interactions highlighted in bold were identified using both SCNIC and SPIEC-EASI.

Abbreviations: HC, healthy control; MSA, multiple system atrophy; PD, Parkinson's disease; SCNIC: Sparse Cooccurrence Network Investigation for Compositional data; SPIEC-EASI: SParse InversE Covariance Estimation for Ecological Association Inference.

Supplementary Table 8. Significant MetaCyc pathways identified in differential pathway abundance analyses

	MSA vs HC			PD vs HC			MSA vs PD		
ANCOM	Pathway	CLR	W value	Pathway	CLR	W value	Pathway	CLR	W value
DADA2-denoising	**PWY-6478**	-1.32	151	**DHGLUCONATE-PYR-CAT-PWY**	0.15	84	---	---	---
	ARGORNPROST-PWY	0.71	118	**PWY-722**	0.15	84	---	---	---
	**---**	---	---	**P101-PWY**,	0.16	83	---	---	---
	---	---	---	**DENITRIFICATION-PWY**	0.17	81	---	---	---
	---	---	---	**TYRFUMCAT-PWY**	0.22	76	---	---	---
OTU-clustering	**PWY-6478** [a]	-0.85	61	**DHGLUCONATE-PYR-CAT-PWY**	0.24	121	PWY-6478	-0.51	81
	---	---	---	**PWY-722**	0.23	120	---	---	---
	---	---	---	**DENITRIFICATION-PWY**	0.25	116	---	---	---
	---	---	---	**TYRFUMCAT-PWY**	0.29	102	---	---	---
	---	---	---	**P101-PWY** [b]	0.20	86	---	---	---
ANCOM-BC	Pathway	LFC	q value	Pathway	LFC	q value	Pathway	LFC	q value
DADA2-denoising	ARGORNPROST-PWY	0.65	0.005	---	---	---	PWY0-1241	-0.94	0.043
OTU-clustering	---	---	---	---	---	---	HEME-BIOSYNTHESIS-II	-0.94	0.014

	---	---	---	---	---	---	PWY0-1415	-0.89	0.024
	---	---	---	---	---	---	PWY-5918	-0.89	0.043
	---	---	---	---	---	---	LPSSYN-PWY	-0.86	0.010
ALDEx2	Pathway	Effect	q value ^{Wi}	Pathway	Effect	q value ^{Wi}	Pathway	Effect	q value ^{Wi}
DADA2-denoising	ARGORNPROST-PWY	0.81	0.022	---	---	---	---	---	---
OTU-clustering	---	---	---	---	---	---	ARGORNPROST-PWY [c]	0.70	0.021
MaAsLin 2	Pathway	Coefficient	q value	Pathway	Coefficient	q value	Pathway	Coefficient	q value
DADA2-denoising	ARGORNPROST-PWY	0.94	0.003	---	---	---	---	---	---
	PWY-6478	-1.86	0.015	---	---	---	---	---	---
OTU-clustering	---	---	---	---	---	---	---	---	---

In ANCOM, the W value represents the number of tests showing significant differences in the ratios of a particular pathway and the other pathway between the two study groups. The CLR represents the difference in the means of centered log-ratio-transformed relative abundance of a pathway between the two study groups. In ANCOM-BC, the LFC quantifies the effect of study group on the bias-corrected absolute abundance of a particular pathway. The q values were determined using the Holm-Bonferroni method. In ALDEx2, the "effect" is determined by calculating the median difference in the CLR-transformed probabilities between the study groups and dividing it by the maximum difference in the CLR-transformed probabilities within the study groups through 128 Monte Carlo samplings. The q value for the Wilcoxon rank test was determined using the Benjamini-Hochberg procedure and is represented as q value ^{Wi}. In MaAsLin 2, the "coefficient" refers to the effect size in the linear model, representing the difference between categorical variables. The q

values were determined using the Benjamini-Hochberg procedure. A positive CLR/ LFC/ “effect”/ “coefficient” suggests that the pathway is more abundant in the first study group. The pathways highlighted in bold were identified using both DADA2-denoising and OTU-clustering.

[a] 10 pathways exhibited a significantly greater abundance in MSA, including PWY-722, DHGLUCONATE-PYR-CAT-PWY, CRNFORCAT-PWY, DENITRIFICATION-PWY, PWY-3661, PWY-4722, PWY-5741, PWY-6728, LEU-DEG2-PWY, and PWY-7644.

[b] Other pathways exhibiting a significantly greater abundance in PD included PWY-5741, PWY-7644, PWY-4722, PWY-3661, CRNFORCAT-PWY, LEU-DEG2-PWY, PWY-6728, PWY-5178, PWY-6948, and PWY-7255.

[c] Other pathways exhibiting a significantly greater abundance in MSA included DENOVOPURINE2-PWY, P163-PWY, PWY-5676, PWY-6125, PWY-6471, PWY-6545, PWY-6588, PWY-7184, PWY-7187, PWY-7196, PWY-7197, PWY-7199, PWY-7200, PWY-7210, PWY-7228, PWY-841, PWY0-162, PWY0-166, and UDPNAGSYN-PWY.

Abbreviations: ALDEx2, ANOVA-Like Differential Expression 2; ANCOM, ANalysis of COmposition of Microbiomes; ANCOM-BC, ANalysis of COmposition of Microbiomes with Bias Correction; CLR, centered log-ratio; HC, healthy control; LFC, log fold change; MaAsLin 2, Microbiome Multivariable Associations with Linear Models 2; MSA, multiple system atrophy; OTU, operational taxonomic unit; PD, Parkinson’s disease.

Supplementary Table 9. ARGORNPROST-PWY pathway and PWY-6478 pathway in pairwise MaAsLin 2 comparisons

Pathway	MSA vs HC		PD vs HC		MSA vs PD	
	Coefficient	q value	Coefficient	q value	Coefficient	q value
DADA2-denoising						
ARGORNPROST-PWY	0.94	0.003	0.16	0.836	0.77	0.116
PWY-6478	-1.86	0.015	-0.39	0.642	-1.41	0.116
OTU-clustering						
ARGORNPROST-PWY	0.67	0.081	0.09	0.826	0.60	0.056
PWY-6478	-1.31	0.067	-0.25	0.695	-1.06	0.052

The “coefficient” refers to the effect size in the linear model, representing the difference between categorical variables. A positive “coefficient” suggests that the pathway is more abundant in the first study group. The q values were determined using the Benjamini-Hochberg procedure.

Abbreviations: HC, healthy control; MSA, multiple system atrophy; OTU, operational taxonomic unit; PD, Parkinson’s disease.

Supplementary Table 10. Significant MetaCyc pathways identified in pairwise multivariable ANCOM-BC comparisons

	MSA vs HC			PD vs HC			MSA vs PD		
	Pathway	LFC	q value	Pathway	LFC	q value	Pathway	LFC	q value
Model 1	DADA2-denoising								
	GLUCARGALACTSUPER-PWY	-1.54	0.016	---	---	---	---	---	---
	GALACTARDEG-PWY	-1.54	0.016	---	---	---	---	---	---
	OTU-clustering								
	---	---	---	---	---	---	HEME-BIOSYNTHESIS-II	-0.91	0.027
	---	---	---	---	---	---	PWY0-1415	-0.87	0.043
	---	---	---	---	---	---	LPSSYN-PWY	-0.83	0.035
Model 2	DADA2-denoising								
	**PWY-6478**	-1.56	0.025	---	---	---	---	---	---
	ARGORNPROST-PWY	0.74	0.027	---	---	---	---	---	---
	OTU-clustering								
	**PWY-6478**	-1.02	0.012	---	---	---	---	---	---

Model 1 adjusted for constipation status. Model 2 adjusted for age, sex, hypertension, constipation status, medications for constipation, and usage of probiotics. The LFC quantifies the effect of study group on the bias-corrected absolute abundance of a particular pathway. A positive LFC suggests that the pathway is more abundant in the first study group. The q values were determined using the Holm-Bonferroni method. Pathways highlighted in bold were identified using both DADA2-denoising and OTU-clustering.

Abbreviations: HC, healthy control; LFC, log fold change; MSA, multiple system atrophy; OTU, operational taxonomic unit; PD, Parkinson's disease.

Supplementary Table 11. Significant MetaCyc pathways identified in pairwise multivariable MaAsLin 2 comparisons

	MSA vs HC			PD vs HC			MSA vs PD		
	Pathway	Coefficient	q value	Pathway	Coefficient	q value	Pathway	Coefficient	q value
Model 1	DADA2-denoising								
	ARGORNPROST-PWY	0.99	0.020	---	---	---	---	---	---
	GALACTARDEG-PWY	-3.37	0.044	---	---	---	---	---	---
	GLUCARGALACTSUPER-PWY	-3.37	0.044	---	---	---	---	---	---
	PWY-6478	-1.94	0.044	---	---	---	---	---	---
	OTU-clustering								
	---	---	---	---	---	---	---	---	---
Model 2	DADA2-denoising								
	ARGORNPROST-PWY	1.16	0.021	---	---	---	---	---	---
	OTU-clustering								
	---	---	---	---	---	---	---	---	---

Model 1 adjusted for constipation status. Model 2 adjusted for age, sex, hypertension, constipation status, medications for constipation, and usage of probiotics. The “coefficient” refers to the effect size in the linear model, representing the difference between categorical variables. A positive “coefficient” suggests that the pathway is more abundant in the first study group. The q values were determined using the Benjamini-Hochberg procedure. Pathways highlighted in bold were identified using both DADA2-denoising and OTU-clustering.

Abbreviations: HC, healthy control; MSA, multiple system atrophy; OTU, operational taxonomic unit; PD, Parkinson’s disease.

Supplementary Table 12. Crucial genera in random forest classifiers for constipation status

MSA		PD		HC	
Genus	Importance score	Genus	Importance score	Genus	Importance score
DADA2-denoising					
***Eubacterium hallii* group**	0.121	***Eubacterium***	0.066	*Phascolarctobacterium*	0.104
Lachnospiraceae	0.067	*Phascolarctobacterium*	0.056	---	---
---	---	*Holdemanella*	0.053	---	---
OTU-clustering					
***Eubacterium hallii* group**	0.050	***Eubacterium***	0.068	*Agathobacter*	0.112
---	---	---	---	*Eggerthella*	0.112

Genera with higher importance scores demonstrated greater efficacy in distinguishing constipation status. The importance scores were determined by the scikit-learn learning estimator. Genera with importance scores ≥ 0.05 are displayed. The genera highlighted in bold were identified using both DADA2-denoising and OTU-clustering.

Abbreviations: HC, healthy control; MSA, multiple system atrophy; OTU, operational taxonomic unit; PD, Parkinson's disease.

Supplementary Table 13. AUCs of random forest classifiers for constipation status built in HC

MSA	PD	HC
DADA2-denoising		
0.30	0.57	0.77
OTU-clustering		
0.27	0.53	0.78

Abbreviations: HC, healthy control; MSA, multiple system atrophy; OTU, operational taxonomic unit; PD, Parkinson’s disease.
